# Bimolecular recombination in methylammonium lead triiodide perovskite is an inverse absorption process

**DOI:** 10.1038/s41467-017-02670-2

**Published:** 2018-01-18

**Authors:** Christopher L. Davies, Marina R. Filip, Jay B. Patel, Timothy W. Crothers, Carla Verdi, Adam D. Wright, Rebecca L. Milot, Feliciano Giustino, Michael B. Johnston, Laura M. Herz

**Affiliations:** 1 Department of Physics, University of Oxford, Clarendon Laboratory, Parks Road, Oxford, OX1 3PU UK; 2 Department of Materials, University of Oxford, Parks Road, Oxford, OX1 3PH UK

## Abstract

Photovoltaic devices based on metal halide perovskites are rapidly improving in efficiency. Once the Shockley–Queisser limit is reached, charge-carrier extraction will be limited only by radiative bimolecular recombination of electrons with holes. Yet, this fundamental process, and its link with material stoichiometry, is still poorly understood. Here we show that bimolecular charge-carrier recombination in methylammonium lead triiodide perovskite can be fully explained as the inverse process of absorption. By correctly accounting for contributions to the absorption from excitons and electron-hole continuum states, we are able to utilise the van Roosbroeck–Shockley relation to determine bimolecular recombination rate constants from absorption spectra. We show that the sharpening of photon, electron and hole distribution functions significantly enhances bimolecular charge recombination as the temperature is lowered, mirroring trends in transient spectroscopy. Our findings provide vital understanding of band-to-band recombination processes in this hybrid perovskite, which comprise direct, fully radiative transitions between thermalized electrons and holes.

## Abstract (web summary)

Radiative bimolecular processes will dominate charge-carrier recombination in hybrid perovskite solar cells operating near the Shockley-Queisser limit. Here, the authors show that such processes are the inverse of absorption and increase as distribution functions sharpen towards lower temperatures.

## Introduction

The past 5 years have seen intense research into the nature of hybrid lead halide perovskites used as active layers in photovoltaic devices^1–3^. Improvements in fabrication processes have led to rapid increases in light-to-electrical power conversion efficiencies beyond 22%, placing these semiconductors at the centre of potentially cheap and sustainable energy production^4,5^. Such rapid advances have brought into question the fundamental physical mechanisms that set the ultimate limits to essential properties such as charge-carrier mobilities^6^, exciton binding energies^7^ and intrinsic (bimolecular) recombination between electrons and holes.

At the heart of a solar cell is the absorbing layer, which is the only layer by design in which incident light should excite charge carriers. Following absorption of photons, excited electrons and holes are only of use if they can be extracted at electrodes to contribute to a photocurrent. Such extraction is impeded by competing charge-carrier recombination processes, which can occur through a variety of mechanisms^8^. The fundamental physical processes governing the dynamics of charge carriers in the absorbing layer hence play a dominant role in the conversion process of photons into storable energy.

Previous transient spectroscopic experiments have revealed the value of rate constants associated with different charge-recombination processes in hybrid perovskites, based on the measurements of transient absorption^9–12^, photoluminescence^13–15^ and terahertz photoconductivity^16,17^ dynamics. The significance of each process varies with the density of charge carriers in the material and thus the intensity of incident light. Under low-level illumination, monomolecular (Shockley–Read–Hall^18^) processes mediated by trap sites such as elemental vacancies, substitutions or interstitials^19^ will dominate charge-carrier recombination. However, with increasing charge-carrier density, bimolecular (radiative) recombination between unbound electrons and holes will begin to take over, while at even higher densities, many-body Auger recombination will contribute^2^.

Any analysis of charge-carrier recombination mechanisms therefore has to be placed in the context of solar irradiation levels (AM1.5) at which Auger recombination was found insignificant^17^. Instead, charge recombination under AM1.5 results from a trade-off between trap-related monomolecular and intrinsic bimolecular electron-hole recombination^2^, with the latter dominating when monomolecular recombination lifetimes exceed 10 μs. Reductions in trap concentration through rapidly improving material processing protocols mean that this limit is already being approached^20,21^, opening the prospect of single-junction solar cells operating near the Shockley–Queisser limit^22^.

Such developments have brought into sharp focus the fundamental nature of bimolecular recombination between electrons and holes in the continuum states of the conduction and valence bands, which will operate in defect-free metal halide perovskites under solar illumination. Despite the importance of this process, relatively little is known about its mechanism to date. While studies have shown bimolecular recombination in hybrid perovskites to be highly non-Langevin^16,23–26^, there is currently no consensus on suitable alternative models. Some studies have suggested that direct band-to-band transitions govern bimolecular recombination, including fully theoretical work, which however currently cannot account for the complexities of Coulomb correlations^27^, and experimental work examining the switch-over point of luminescence intensity between the trap-mediated recombination regime at low charge-carrier densities, and the bimolecular regime at higher densities^28^. However, other studies have hypothesised a range of more exotic influences, including: Rashba spin-orbit coupling^29^, a mixture of indirect and direct band gap states^30^, and relativistic effects^31^. Identification of the correct mechanism can be further complicated by the potential presence of electronic transitions between multiple and non-parabolic bands and an energy dependence of the associated matrix elements, which have not been evaluated with certainty to date.

In this work, we show unambiguously that bimolecular electron-hole recombination in lead iodide perovskites can be understood in terms of the inverse process to absorption, analogous to the case of GaAs^32–34^ and silicon^35,36^. With the help of insights gained from ab initio calculations regarding the validity and applicatibility of Elliott’s theory to CH_3_NH_3_PbI_3_, we are able to correctly unravel the contributions to absorption from bound electron-hole pairs (excitons) from those of unbound electrons and holes in the continuum states of the semiconductor. This approach allows us to demonstrate a clear correlation between the temperature-dependent changes in absorption spectra of charge carriers and the rate of bimolecular charge-carrier recombination.

## Results

### Absorption modelling

To probe whether bimolecular recombination between unbound electrons and holes is the inverse of absorption, we need to separate accurately the contributions to the absorption from continuum states and from Coulombically bound electron-hole pairs (excitonic states). Such separation is usually achieved through Elliott’s theory^37^, however, previous attempts for metal halide perovskites have found significant deviations from experimental data for this theory at energies sufficiently above the band gap^12,38,39^, which calls into question the rationale for using this model. Below, we perform an analysis of absorption spectra for the most frequently investigated metal halide perovskite, CH_3_NH_3_PbI_3_, in combination with high-accuracy GW ab initio calculations to unravel the causes of such effects.

**Fig. 1:**
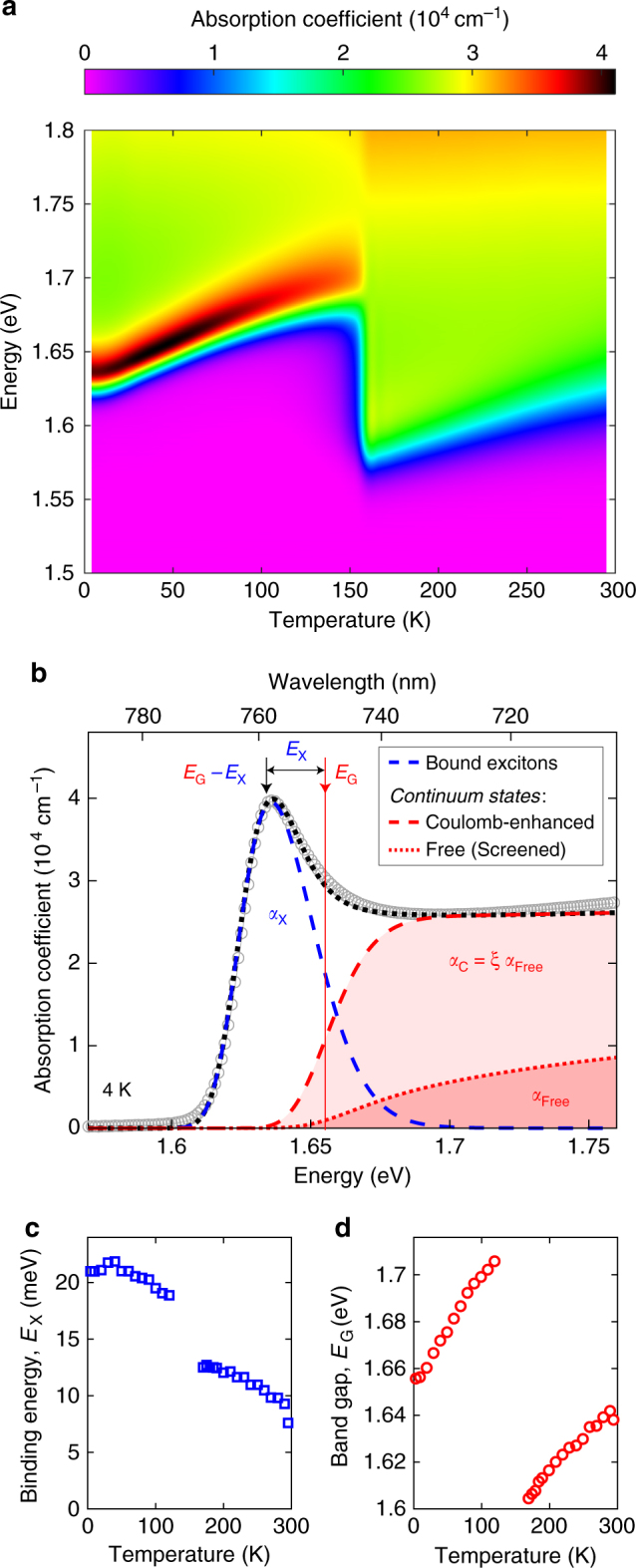
Absorption modelling of CH_3_NH_3_PbI_3_. **a** Two-dimensional colour map of the absorption spectra of CH_3_NH_3_PbI_3_ as a function of energy and temperature. **b** Absorption spectrum of CH_3_NH_3_PbI_3_ taken at 4 K. The black dotted line is a fit to the data based on Elliott’s theory as described in the main text, which allows the separation of the contributions to the total absorption coefficient arising from bound excitonic states (*α*_X_, blue dashed line) and Coulomb-enhanced electron-hole continuum states (*α*_C_, red dashed line), which is increased beyond the absorption from free (screened) electrons and holes (*α*_Free_, red dotted line) by the Coulomb-enhancement factor *ξ*. Arrows indicate the position of the band gap (*E*_G_) and the value of the exciton binding energy (*E*_X_). **c** Exciton binding energy and **d** band gap energy of CH_3_NH_3_PbI_3_ as a function of temperature, obtained from fits based on Elliott’s theory to the band-edge absorption data shown in Figure S3 in Supplementary Information. The small gap in displayed points between 140 and 170 K is owing to the coexistence of the orthorhombic (<140 K) and tetragonal (>170 K) phases^69^ for this temperature range, which leads to unreliable fits to the absorption edge in this region

To determine the temperature-dependent absorption spectra of CH_3_NH_3_PbI_3_ (Fig. 1a), reflection-transmission measurements were performed on thin films by use of a Fourier transform infrared (FTIR) spectrometer. Thin films were fabricated through dual-source vapour deposition (Methods), which yields smooth, uniform surfaces^40^, as evidenced from the observation of Fabry–Perot oscillations (Supplementary Fig. 1b) and sharp absorption onsets devoid of scattering effects that would otherwise complicate the modelling. Figure 1b shows the absorption spectra of CH_3_NH_3_PbI_3_ at 4 K along with a fit based on Elliott’s theory^37^ within the first 100 meV above the band edge (full details are included in Supplementary Note 5). Within this energy range, this theory is found to reproduce accurately the spectral shape of the absorption, however, at higher energies significant deviations occur.

Elliott’s theory accounts for the enhancement of light absorption through Coulombic attraction between electrons and holes in the conduction and valence bands of a semiconductor^37^. As Fig. 1b illustrates, such interactions significantly increase the absorption coefficient near the band gap energy *E*_G_ beyond that expected for a free electron-hole picture. Here, Coulombic attraction leads to additional absorption into bound excitonic states (*α*_X_(*E*), blue dashed line) that manifest themselves as a series of hydrogenic peaks at energies below *E*_G_. Importantly, even above *E*_G_, Coulomb interactions substantially enhance the absorption coefficient for electron-hole continuum states (*α*_C_(*E*), red dashed line), by a factor *ξ*(*E*) beyond that expected for free electrons and holes (*α*_Free_(*E*), dotted line). *ξ*(*E*) is the Coulomb-enhancement factor derived from the values of the band gap and binding energy (see Eq. (11) in the Supplementary Information) and is therefore not a free (independent) parameter of the model. As a result, the continuum absorption spectrum differs from the classical square-root dependence expected for uncorrelated charges in a direct semiconductor (*α*_Free_), resulting instead in a step-like onset at the band gap (*α*_C_)^37,39^.

The correct disentanglement of these excitonic and continuum states is key to unravelling the mechanisms of bimolecular recombination, which arises only from interactions between unbound electrons and holes. As discussed in more detail below, we also need to account for the possibility that the Coulomb interactions between charge carriers are screened, which may occur at sufficiently high charge-carrier densities (above the Mott transition^41^). In addition, our model accounts for spectral broadening caused by electron–phonon interactions and energetic disorder^42^ through convolution with a suitable function (Supplementary Note 5). We note that photon absorption also depends on the electric dipole matrix element, which is assumed to be independent of photon energy in Elliott’s theory^37^ and hence in our modelling of the experimental data as shown in Fig. 1.

A correct determination of the exciton binding energy *E*_X_ is a prerequisite for the accurate extraction of the continuum states. We determine the value of *E*_X_ to be 20 ± 2 meV for the low-temperature (0–160 K) orthorhombic phase from fits based on Elliott’s theory, which is in good agreement with the low-temperature value of 16 ± 2 meV determined from magneto-absorption studies^7^. Figure 1c shows that the binding energy declines only slightly with temperature within each structural phase, apart from a rapid drop by ~5 meV that occurs at the phase transition into the tetragonal structure. These changes can be understood in terms of temperature-dependent trends in the dielectric function at low frequencies; in particular, at the orthorhombic-tetragonal phase transition a step-change in permittivity^43^ and hence exciton binding energy occurs. The trends with temperature of the binding energy are similar to those reported in previous studies^44–46^ and the value of *E*_X_ ~8 meV at room temperature is commensurate with efficient exciton dissociation, as suggested in other studies^7,16,44,45,47,48^.

Careful modelling of the band-edge absorption also allows us to extract values for the band gap energy that can be compared with those determined from ab initio calculations discussed below. Figure 1d illustrates that the extracted band gap energy *E*_G_ generally increases with temperature, in contrast with trends for typical inorganic semiconductors such as GaAs^49^. This phenomenon has previously been attributed to a reverse ordering of the band-edge states^1,50–52^. At the transition between the orthorhombic and tetragonal phases (160 K), *E*_G_ displays a noticeable discontinuity of 100 meV as previously observed^17^ and deriving from changes in octahedral tilts^53^.

### Theoretical calculations

As mentioned above, the use of Elliott’s theory to model the absorption edge of CH_3_NH_3_PbI_3_ has proven to be controversial, given that several studies (including the present) found that satisfactory agreement could only be obtained for energies near the band edge^12,38,39^. It has been postulated that non-parabolicity of the conduction and valence bands could be to blame^28,44,54^, however, this would have to be severe in order to account for the observed discrepancies. Here we resolve this controversy by showing that the divergence mostly arises from a breakdown of several assumptions that Elliott’s theory is based on, which are that (i) only one valence and one conduction band are involved in the transitions; (ii) the bands are spherical (i.e. parabolic and isotropic); and (iii) the electric dipole transition matrix element is independent of photon energy. We examine the validity of these assumptions in the context of CH_3_NH_3_PbI_3_ by conducting highly accurate first-principles calculations that can take into account potential non-parabolicity and higher-lying electronic transitions.

**Fig. 2:**
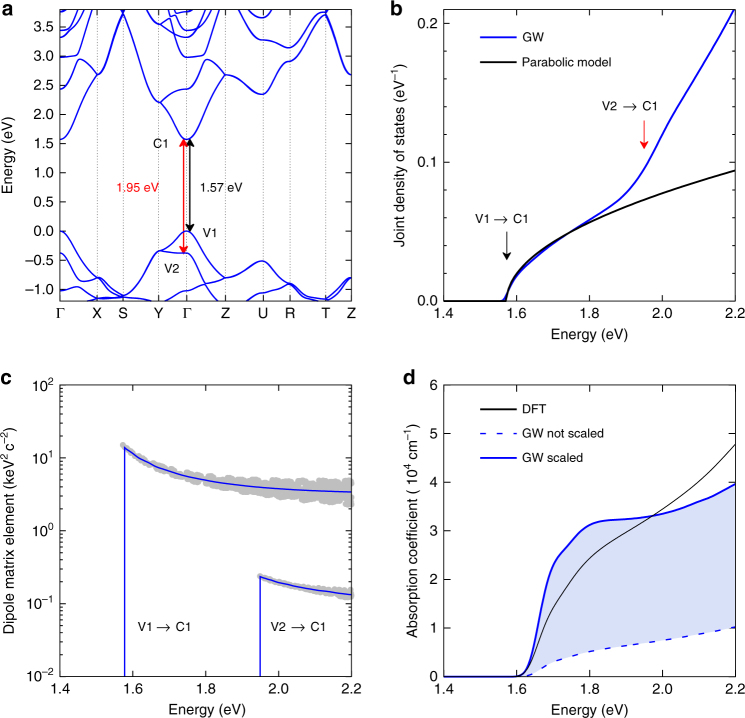
First-principles calculations of CH_3_NH_3_PbI_3_. **a** Quasi-particle band structure of CH_3_NH_3_PbI_3_ calculated using the GW approximation (see Supplementary Information for details). The black arrowed line shows the direct band transition from the first valence band (V1) to the first conduction band (C1) at an energy of 1.57 eV. The red arrowed line shows the transition from the second valence band (V2) to C1, at an energy of 1.95 eV. **b** The joint density of states derived from GW calculations (blue line), compared with the function expected from a simple parabolic band model (black line). The two arrows mark the two lowest energy transitions corresponding to those marked in **a**. **c** The electric dipole transition matrix element for the V1 ↔ C1 and V2 ↔ C1 transitions. Grey filled circles represent the matrix element as calculated at different *k*-points in the Brillouin zone using Eq. (3) of the Supplementary Information. The blue lines show the value averaged over all directions in the Brillouin zone. **d** Absorption coefficient spectrum calculated from density-functional theory (DFT) calculations (black), and GW calculations (blue) within the independent-particle approximation (i.e., without considering electron-hole Coulomb interactions). We report results using scaled (solid) and unscaled (dashed) dipole matrix elements, as derived using Eqs. (4) and (5) given in the Supplementary Information. The light blue region represents the uncertainty in the magnitude of the optical absorption spectrum owing to the approximate calculation of the dipole matrix elements. The functions have been shifted along the energy axis to make the onset coincide with the experimental value of *E*_G_

Figure 2a shows the electronic band structure of CH_3_NH_3_PbI_3_ calculated from first principles using the GW approximation. Details for all calculations can be found in Supplementary Note 4 and in previous articles^55,56^. We calculate a quasi-particle band gap (V1 ↔ C1) of 1.57 eV, which is in good agreement with the band gap energy we determine at 4 K (1.66 eV, see Fig. 1d). The small difference between the calculated and measured band gaps derives from the absence of electron–phonon interactions in our calculations. Interestingly, our calculations also reveal the presence of another electronic transition (V2 ↔ C1) with an onset energy of 1.95 eV, which is relatively close to the band edge and therefore has the potential to influence the shape of the absorption onset.

From the calculated GW electronic band structure, we are also able to extract the quasi-particle electron and hole effective masses, as shown in Table 1. Effective masses are calculated as the inverse of the second derivatives of the energy with respect to the wave vector at the Γ-point, with derivatives evaluated numerically from finite differences. The reduced effective mass of 0.11*m*_e_ is in excellent agreement with experimental values^7^. The accuracy of these calculations in terms of reproducing closely the experimental values of both band gap energy and effective masses highlights the power of the GW method, in comparison with traditional density-functional theory (DFT) that tends to underestimate effective masses and band gap energies (Supplementary Note 4).

**Table 1:** Comparison between calculated and experimental band gap energies and effective masses

	Theory (GW)	Experiment
*E*_G_ (eV)	1.57	1.66
$m_{\mathrm{h}}^ \ast$	0.23	—
$m_{\mathrm{e}}^ \ast$	0.22	—
*μ*	0.11	0.104 ± 0.003^7^

Effective masses are given in units of the electron rest mass. The values of the electron and hole effective masses were obtained as isotropic averages of the diagonalized effective mass tensor at the Γ-point

*E*_G_ band gap at 4 K, $m_{\mathrm{e}}^ \ast$ electron (C1) effective mass, $m_{\mathrm{h}}^ \ast$ hole (V1) effective mass,* μ* = (($m_{\mathrm{e}}^ \ast$)^−1^ + ($m_{\mathrm{h}}^ \ast$)^−1^)^−1^ reduced effective mass

With a conclusive computational framework in place, we probe the possibility of non-parabolicity contributing to deviations from Elliott’s theory. We compute the joint density of states using the quasi-particle eigenvalues and contrast this with the trend expected for a simple parabolic onset based on the calculated reduced effective mass. As Fig. 2b shows, the density of states associated with the V1 ↔ C1 transition (onset 1.57 eV) is remarkably parabolic for photon energies of up to ~250 meV above the gap. However, the second transition (1.95 eV; V2 ↔ C1) makes a significant contribution to the joint density of states and will give rise to additional absorption, if it has significant oscillator strength. Hence, we conclude that the parabolic approximation assumed in Elliott’s model will hold for photon energies within ~250 meV of the band edge, but additional electronic transitions will lead to deviations already within the first ~200 meV.

In addition, we examine the validity of the assumption that the electric dipole transition matrix element is constant with photon energy in CH_3_NH_3_PbI_3_. It has long been known for inorganic semiconductors such as silicon or germanium that significant deviations may occur from this simplifying approximation^57^. We therefore calculate the matrix element at a number of different *k*-points in the Brillouin zone relating to different energy transitions (Fig. 1c). Grey circles are the calculated values at different *k*-points with the average value shown as a blue line. Our results clearly demonstrate that the matrix element decreases significantly with increasing energy for both the V1 ↔ C1 and V2 ↔ C1 transitions; therefore Elliott’s approximation of constant matrix element will only hold close to the band edge. In addition, we find that the magnitude of the V2 ↔ C1 transition is approximately 1 order of magnitude lower than that of the V1 ↔ C1 transition, which means that its contribution to absorption above 1.95 eV is not as significant as in the unweighted joint density of states.

Our rigorous evaluation of all factors contributing to the absorption allows us to derive the absorption coefficient entirely from ab initio calculations (Supplementary Note 4), as shown in Fig. 2d for both DFT and GW approximations. An accurate calculation of the absorption coefficient within the GW approximation requires the inclusion of the off-diagonal terms of the GW self-energy, which is computationally prohibitive for the case of CH_3_NH_3_PbI_3_. Instead, in Fig. 2d we show two limits of the GW absorption spectra obtained using the dipole matrix elements calculated from DFT, both before and after scaling with respect to the quasi-particle transition energies (Supplementary Information). Figure 2d shows that the absorption coefficient is sensitive to scaling, and can vary by up to a factor of 4, while the shape of the spectrum does not vary significantly. Moreover, it can be shown that the f-sum rule in these two cases is either overestimated, if the matrix elements are scaled, or underestimated in the unscaled case, consistent with the respective magnitudes of the absorption coefficient shown in Fig. 2d. For this reason, we propose that the true absorption coefficient is bracketed by the scaled and unscaled GW spectra, which give good agreement with the experimental data (*α*_Free_, Fig. 1b). This result indicates that the inclusion of band non-parabolicity, higher-lying transition and the functional dependence of the matrix element are fundamental for the accurate evaluation of the absorption coefficient in CH_3_NH_3_PbI_3_. Hence, these ab initio calculations have enabled us to individually explore each assumption that Elliott’s theory is built on, allowing us to probe its validity for hybrid perovskite semiconductors. Overall, our analysis conclusively reveals that deviations from Elliott’s theory for CH_3_NH_3_PbI_3_ are a reflection of a breakdown in the assumptions the model is based on, for energies somewhat higher than the band edge. Our evaluation of these factors however suggest that within ~100 meV, the simplified assumptions of Elliott’s theory holds, allowing us to perform an analysis of band-edge charge-carrier recombination based on the continuum states extracted from this model.

### Bimolecular recombination

**Fig. 3:**
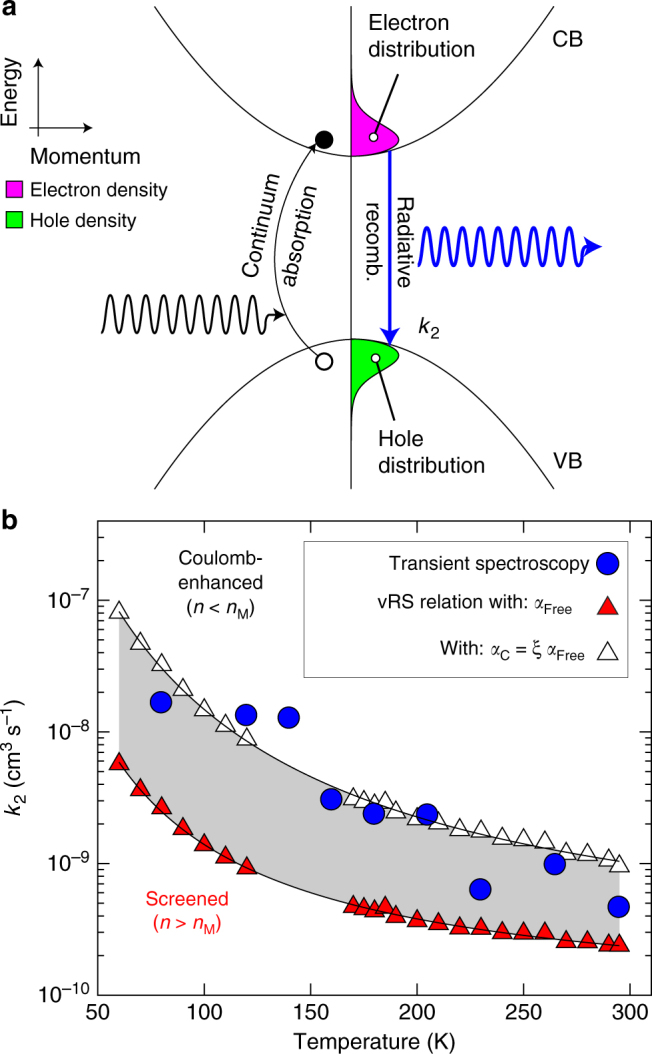
Temperature dependence of bimolecular recombination in CH_3_NH_3_PbI_3_. **a** Schematic diagram of the energy dispersion relation and charge-carrier distributions, which have a determining role in the bimolecular charge-carrier recombination process. The purple and green filled areas show the Fermi–Dirac distributions of the excited electrons and holes in the conduction and valence bands, respectively. **b** Bimolecular recombination rate constant (*k*_2_) as a function of temperature, displayed on a semi logarithmic scale. Triangles show *k*_2_ as deterimined through the van Roosbroeck and Shockley relation from fits to the experimentally obtained absorption spectra, based on either the electron-hole continuum absorption coefficient including Coulomb interactions (*α*_C_, white triangles), or the free (screened) electron-hole coefficient (*α*_Free_, red triangles), with the former applicable at low charge-carrier densities (*n* < *n*_M_) and the latter for charge-carrier densities exceeding the Mott density *n*_M_. Points were fitted with phenomenological expressions (solid line) that are listed in Supplementary Information. Blue circles show values of *k*_2_ obtained previously^17^ from transient terahertz photoconductivity spectroscopy that monitors free-charge carrier recombination dynamics, as corrected for internal self-absorption effects^62^ to yield intrinsic values of *k*_2_ (see Supplementary Information for details)

With a comprehensive picture of the contributions to the absorption onset in place, we proceed to link bimolecular recombination of electrons and holes to the reverse process of light absorption by continuum states. Figure 3a shows a schematic diagram linking the Fermi–Dirac distributions of electrons and holes with absorption and emission processes as mediated by the Bose–Einstein functions of the photon radiation field. To validate such links, we first evaluate the temperature-dependent bimolecular recombination rate constants that would be expected according to the van Roosbroeck–Shockley^58^ relation for absorption by electrons and holes in the presence and absence of Coulomb correlations. We then compare the calculated rate constants with those obtained from transient spectroscopy, showing excellent agreement for both absolute values and temperature trends.

The theory of photon-radiative recombination of electrons and holes was first developed by van Roosbroeck and Shockley^58^ and applies the principle of detailed balance to the thermal equilibrium rate of photon absorption, i.e., it assumes the rate of radiative recombination at thermal equilibrium at a certain frequency to be equal to the rate of generation of electron-hole pairs by thermal radiation at the same frequency (Fig. 3a)^59^. The van Roosbroeck–Shockley relation hence allows deduction of the radiative recombination rate and bimolecular recombination rate constant from steady-state absorption spectra. The theory is based on the assumption of unit quantum efficiency and is applicable to a material in which there is no *k*-selection rule, or to a non-degenerate material in which there is a selection rule^32,60^, and has extensively been applied to group IV, VI, and III–V direct and indirect semiconductors in the past^60^.

The van Roosbroeck–Shockley relation describes the temperature-dependent radiative recombination rate *R*_rad_(*T*) by the following function^58^:

1 $$R_{{\mathrm{rad}}}(T) = {\int}_{\!\!\!\!0}^\infty \rho (E,T)P(E,T)\mathrm{d}E,$$

where *ρ*(*E*, *T*)d*E* is the density of photons in the material in the interval d*E*, and *P*(*E*, *T*) is the probability per unit time that a photon of energy *E* is absorbed and hence is proportional to the absorption coefficient. The principle of detailed balance demands that transitions between any two states occurs with equal frequency and in either direction at equilibrium and also prevents the maintenance of equilibrium by means of cyclic processes^61^. Hence, the absorption coefficient that enters Eq. (1) is that of the continuum states and the choice of whether to include Coulombic enhancement is discussed below. Dividing the radiative recombination rate by the square of the intrinsic carrier concentration, *n*_i_, we are able to obtain the bimolecular recombination rate constant, $k_2(T) = R_{{\mathrm{rad}}}(T){\mathrm{/}}n_{\mathrm{i}}^2(T)$, which is also often referred to as the ‘B-coefficient’ in literature on inorganic semiconductors. All associated equations are detailed in full in Supplementary Note 6. The intrinsic carrier concentration and the photon distribution are dependent on the Fermi–Dirac and Bose–Einstein distributions respectively, which vary strongly with temperature.

The correct evaluation of *k*_2_ through the van Roosbroeck–Shockley relation requires a choice to be made about which absorption coefficient spectrum enters, Eq. (1), as discussed in detail in Supplementary Note 6. Past studies for the case of silicon^35,36^ discussed the role of Coulomb interactions for the radiative recombination between electrons and holes in the conduction and valence band. In the presence of Coulomb correlations, absorption is strengthened by the energy-dependent factor *ξ* and the Coulomb-enhanced spectrum *α*_C_(*E*) may be used, while in the absence of such interactions, *α*_Free_(*E*) may be used in Eq. (1). In general, Coulomb correlations increase the probability of an electron and a hole to exist in the same space, and therefore such interactions should a priori be included in a calculation of *k*_2_. However, at charge-carrier densities *n*, exceeding the Mott density *n*_M_, Coulomb interactions will be effectively screened by the presence of the high background charge-carrier density, therefore the free electron-hole absorption *α*_Free_ is the relevant quantity. The Mott density *n*_M_ places a quantitative value on the transition from an insulating gas of excitons at lower densities to a metal-like state of an electron-hole plasma at high charge-carrier densities^41^. The electrostatic force between charged particles is a continuous function of their relative distances to each other, therefore it is intrinsically challenging to define a distinct density at which a Mott transition occurs. Hence, a wide range of expressions for the Mott density have been proposed, which depend on different model assumptions and complexities, and whose validity is also temperature-dependent. A detailed discussion and literature summary for a variety of models can be found in the Supplementary Note 7. To represent the variety of such models, we evaluate the Mott density as a function of temperature^41^, based on different expressions and using values for the exciton binding energy shown in Fig. 1c and the reduced effective mass obtained from GW calculations (Table 1), as described in Supplementary Note 7. We find values of *n*_M_ to be distributed about 10^17^ cm^−3^ for 50 < *T* < 295 K.

Figure 3b displays the temperature dependence of the bimolecular recombination rate constant *k*_2_ derived from the unbound electron-hole absorption coefficient spectra through the van Roosbroeck–Shockley relation, for the case of Coulomb-enhanced absorption (*α*_C_(*E*), white triangles) and screened Coulomb interactions (*α*_Free_(*E*), red triangles), i.e., above and below the Mott transition. At room temperature, Coulomb enhancement of the continuum absorption increases the bimolecular recombination constant by about a factor 4 with respect to the case of screened interactions. This discrepancy increases with decreasing temperature as Coulomb-enhancement effects become more prominent. For both cases, the value of *k*_2_ increases by roughly 1 order of magnitude between room temperature (295 K) and 50 K. Our analysis reveals that this increase is directly linked with the sharpening of the Fermi–Dirac and Bose–Einstein distribution functions with decreasing temperature, with changes in absorption (and hence refractive index) making only a very minor contribution by comparison. As the photon distribution function and the thermal spread of electrons and holes across the conduction and valence bands narrow, bimolecular recombination is substantially enhanced.

To validate our overall approach, we compare these trends with values obtained from terahertz photoconductivity transients, as reported in a previous study^17^ and corrected for self-absorption effects^62–66^ to yield the intrinsic values of *k*_2_ (see Supplementary Note 8 for details). Figure 3b reveals that temperature trends observed in transient spectroscopy agree very well with those derived from the van Roosbroeck–Shockley relation. A comparison in terms of absolute values shows that these transient spectroscopic data are closest to the case of Coulomb-enhanced (unscreened) bimolecular recombination, although most points fall within the intermediate regime (shaded region). This result is not surprising, given that a clear observation of bimolecular processes in transient spectroscopy requires excitation with laser pulse fluences for which these recombination events begin to dominate over trap-related recombination that is prominent at lower charge-carrier densities. As suggested previously^2^ and described in detail in Supplementary Note 6, this case applies at charge-carrier densities above ~10^15^–10^17^ cm^−3^ for typical CH_3_NH_3_PbI_3_, which may potentially come near the Mott density (Figure S7d). However, a recent study^67^ reported the direct observation of excitonic Rydberg states following excitation with fluences 1 order of magnitude higher than the highest fluence utilised for the transients against which we reference our calculated values of *k*_2_ here. We therefore conclude that the most adequate scenario for comparison is with values obtained from the Coulomb-enhanced (unscreened) absorption coefficient, for which Fig. 3b also indicates best agreement. Hence, our analysis clearly supports the notion of an inverse absorption process leading to bimolecular recombination in CH_3_NH_3_PbI_3_.

Our findings have direct impact on the use of hybrid perovskites in photovoltaic applications because they inherently connect the requirement for strong light absorption near the band edge with the presence of significant electron-hole recombination. However, we note that this fundamental link in no way inhibits the efficient operation of photovoltaic devices based on CH_3_NH_3_PbI_3_. While faster bimolecular charge-carrier recombination will shorten charge-carrier diffusion lengths, it also mandates higher absorption coefficients, and hence absorber layers can be made thinner to compensate. Charge-carrier diffusion lengths for thin hybrid perovskite films are already in the micron regime^13^, which exceeds their typical thickness in solar cells of a few hundred nanometres, thus allowing excellent charge-carrier extraction to electrodes. Therefore, while the mechanism we identify here poses a fundamental hurdle to a reduction of bimolecular recombination, it does not preclude the development of high-efficiency photovoltaic cells based on lead halide perovskites. In addition, our findings demonstrate that bimolecular (radiative) recombination rate constants will depend to some extent on light intensity levels. Under solar illumination^2^, charge-carrier densities in CH_3_NH_3_PbI_3_ are near 10^15^–10^16^ cm^−3^ and therefore below the Mott density, leading to Coulomb-enhanced electron-hole recombination. However, implementation in solar concentrators could potentially lead to charge-carrier densities approaching  10^18^ cm^−3^, for which bimolecular recombination rate constants are likely to be reduced as a result of screening.

With the efficiency of perovskite photovoltaic cells rapidly increasing, the Shockley–Queisser limit is not too far from reach. We have examined the physical processes behind bimolecular electron-hole recombination in CH_3_NH_3_PbI_3_, which will dominate over trap-assisted monomolecular recombination once this limit is approached. Our direct comparison between bimolecular recombination rate constants derived from the van Roosbroeck–Shockley relation, with those extracted from transient spectroscopic measurements, demonstrates that these processes can be seen as the inverse to absorption. The established validity of the van Roosbroeck–Shockley relation further demonstrates that photon emission derives from fully radiative^2,28,62^ band-to-band transitions, which in this case involve the recombination of thermalized electrons and holes across a direct band gap. We demonstrate that the sharpening of photon, electron and hole distribution functions enhances the bimolecular radiative rate by roughly an order of magnitude as the temperature is lowered from room temperature to ~50 K. In addition, our highly accurate GW ab initio calculations have clearly identified the salient factors defining the shape of the absorption onset, such as non-parabolicity, higher-lying transitions and the correct functional dependence of the matrix element. We further show that screening of the Coulomb interactions between electrons and holes above the Mott transition will reduce bimolecular recombination constants, meaning that such values have to be considered in the context of the charge-carrier densities present. Our findings thus provide a fundamental understanding of the electronic processes in these hybrid perovskites, which will allow for guided exploration of alternative stoichiometries with desirable photovoltaic properties.

## Methods

### Perovskite thin-film fabrication

Dual-source thermal evaporation was used to grow thin films of CH_3_NH_3_PbI_3_ on z-cut quartz substrates under high vacuum as reported previously^68^. CH_3_NH_3_I and PbI_2_ were placed in separate crucibles, and the substrates were mounted on a rotating substrate holder to ensure a uniform film was deposited. The temperature of the substrates was kept at 20 °C throughout the deposition and the chamber was allowed to reach a high vacuum (10^−6^ mbar). Once the deposition rate for CH_3_NH_3_I and PbI_2_ had stabilised, the substrates were exposed to the vapour for 2 h at a nominal growth rate of 0.6 Å s^−1^. The deposition rates of both CH_3_NH_3_I and PbI_2_ were monitored using a quartz crystal microbalance to ensure that a 1:1 molar ratio was achieved in the final composition of the film. The thickness of the thin-film CH_3_NH_3_PbI_3_ was measured by cross-sectional scanning electron microscope (SEM) images to be 435 ± 5 nm (Supplementary Information Fig. 2).

### Reflection-transmission measurements

A FTIR spectrometer (Bruker Vertex 80v) was used in conjunction with a gas exchange cryostat (Oxford Instruments OptistatCF2) to measure the temperature-dependent reflectance and transmittance of CH_3_NH_3_PbI_3_. The system was set up with a tungsten halogen lamp as the illumination source, a CaF_2_ beamsplitter and a silicon detector. A schematic of the spectrometer, and the reflection and transmission spectra at 4 K are included in Supplementary Note 1.

### Data availability

Data supporting this publication is available from the corresponding author on request. The calculations were performed using the open-source software projects Quantum ESPRESSO, Yambo and Wannier90, which can be freely downloaded from www.quantum-espresso.org, www.yambo-code.org and www.wannier.org, respectively.

## Electronic supplementary material

Supplementary Information
