# Supplementary material for: Bimolecular recombination in methylammonium lead triiodide perovskite is an inverse absorption process
Source: Nat Commun. 2018 Jan 18;9:293. doi: 10.1038/s41467-017-02670-2 (PMC5773627; doi:10.1038/s41467-017-02670-2)
Supplement: Supplementary file 1 — Supplementary Information [file 41467_2017_2670_MOESM1_ESM.pdf]

Supplementary Note 1: Fourier Transform Spectroscopy

A Fourier transform infrared spectrometer (Bruker Vertex 80v) was used in conjunction with a gas exchange cryostat (Oxford Instruments OptistatCF2) to measure the temperature dependent reflectance (R_s) and transmittance (T_s) of $CH_3NH_3PbI_3$. A tungsten halogen lamp source, a calcium fluoride beamsplitter and silicon detector were used. The angle of incident light to the normal of the sample was $\theta = 11°$. Supplementary Figure 1a shows a schematic diagram of the setup used. Supplementary Figure 1b shows the reflection and transmission spectra of the sample at 4 K. Below the band gap (1.3 to 1.6 eV) the sum of the R_s and T_s of the sample is approximately equal to 1, as expected, since light is not absorbed in this region. However, there are large modulations in R_s and T_s which are Fabry-Perot oscillations. This demonstrates the excellent surface quality of the material and indicates negligible scattering of light at the surface which is important for accurate calculation of the absorption spectrum, in particular near its onset.

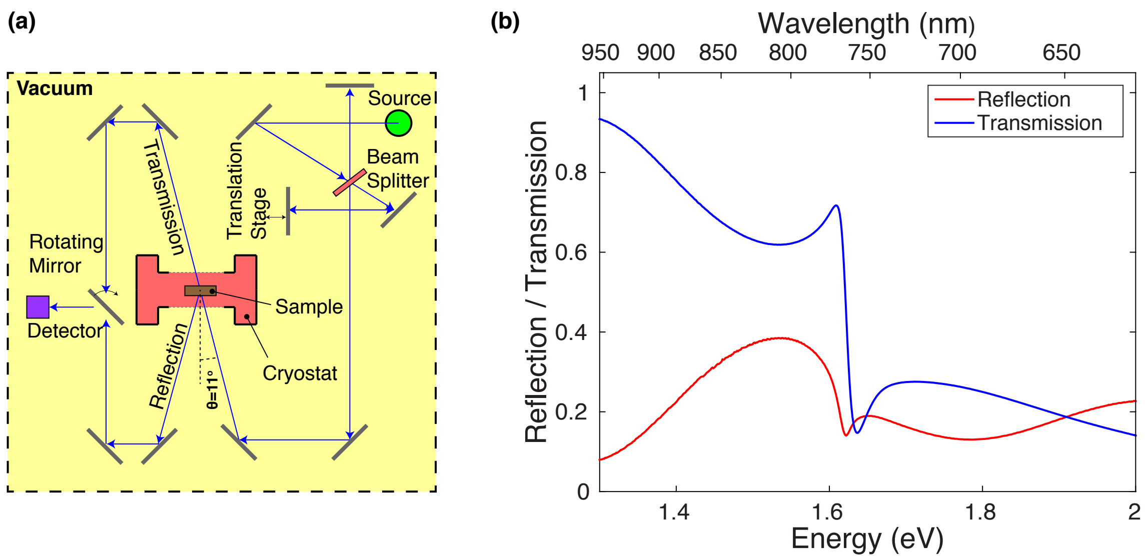

Supplementary Figure 1: Fourier transform infrared spectroscopy. (a) Schematic diagram of the Fourier transform infrared spectrometer with a sample mounted inside a cryostat. (b) Reflection and transmission spectra of $CH_3NH_3PbI_3$ at 4 K.

Supplementary Note 2: Scanning Electron Microscopy

Supplementary Figure 2 shows a scanning electron microscope (SEM; Hitachi S-4300) image of a cross section through the $CH_3NH_3PbI_3$ layer investigated in this study. Based on the analysis of this cross-sectional image, the thickness of the film was determined to be 435 ± 5 nm. The image was taken at a working distance of 9.9 mm and a voltage of 2.0 kV.

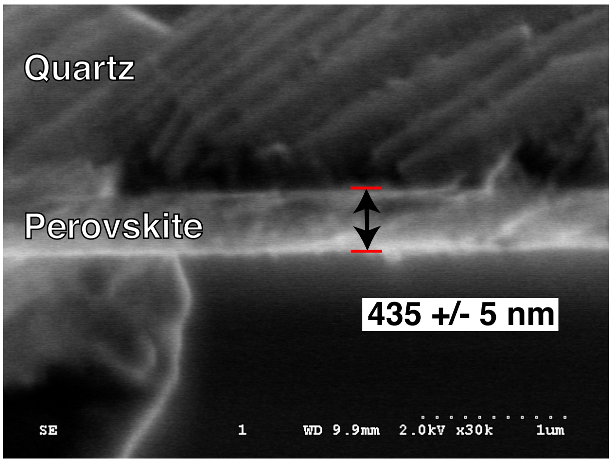

Supplementary Figure 2: Cross-sectional scanning electron microscopy (SEM) image of a $CH_3NH_3PbI_3$ perovskite film, showing a thickness of 435 ± 5 nm.

Supplementary Note 3: Experimentally Determined Absorption Spectra

Absorption coefficient spectra for the full temperature range of $CH_3NH_3PbI_3$ are shown in Supplementary Figure 3. A phase transition occurs in the range 140–170 K between the low-temperature orthorhombic and the tetragonal structures. At 155 K there is clear evidence of a coexistence of the two phases.[1] The excitonic absorption peak is most pronounced at low temperatures. At higher temperatures the peak becomes smeared out because of thermal broadening. The absorption coefficient (α) was obtained from the reflectance (R_s) and transmittance (T_s) by,

$$\alpha = -\frac{1}{d}\ln\left(\frac{T_\mathrm{s}}{1-R_\mathrm{s}}\right) , \tag{1}$$

where d is the thickness of the sample. Supplementary Equation 1 is valid for samples on non-absorbing substrates such as the quartz substrates used in our study.

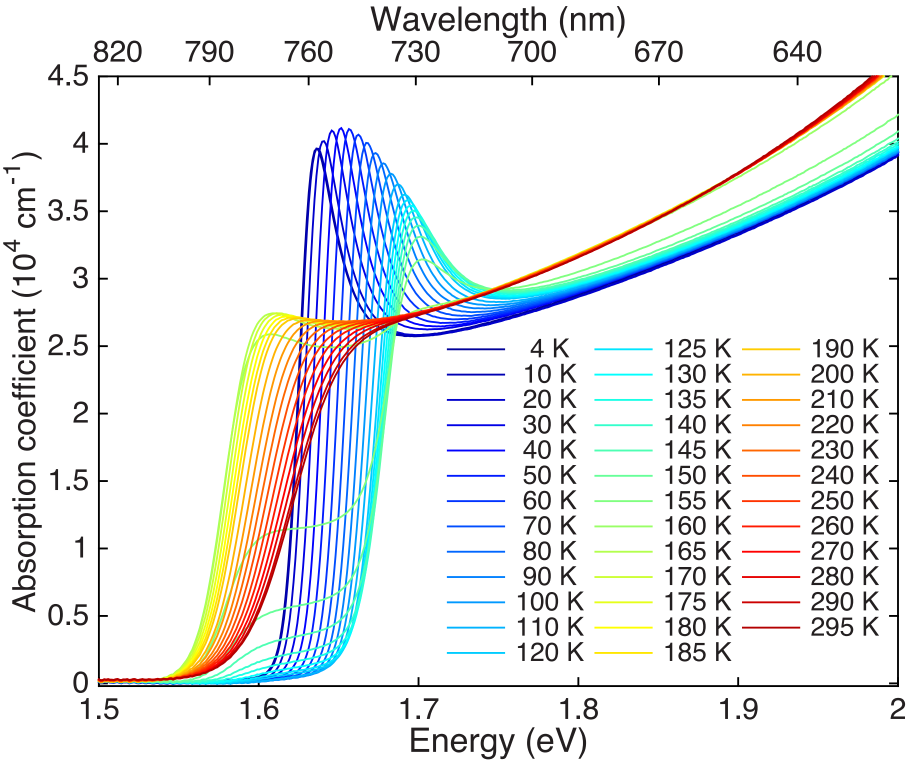

Supplementary Figure 3: Absorption spectra of $CH_3NH_3PbI_3$ for the full temperature range from 4 K to 295 K as a function of energy (eV) on the bottom axis and wavelength (nm) on the top axis. The absorption coefficient was determined using the reflection and transmission spectra and Supplementary Equation 1.

Supplementary Note 4: GW and DFT First Principle Calculations

All calculations are performed for the low-temperature orthorhombic crystal structure of $CH_3NH_3PbI_3$, as reported by Baikie et al.[2] This choice is motivated by the fixed orientation of the $CH_3NH_3^+$ cations in the orthorhombic phase.

We calculate the electronic structure of $CH_3NH_3PbI_3$ within the local density approximation to density functional theory (DFT/LDA)[3,4] including spin-orbit coupling, as implemented in Quantum Espresso.[5] Furthermore, we use the *GW* approximation[6] as implemented in the Yambo code,[7] and a self-consistent scissor *GW* scheme[8,9] to calculate the quasiparticle band structures. We use Wannier interpolation as implemented in the Wannier90 code[10–13] in order to obtain the quasiparticle eigenvalues at arbitrary **k**-points in the Brillouin zone. In the following we give the computational details of every calculation step.

DFT calculations. For the ground state calculations we use norm-conserving Troullier-Martins[14] pseudopotentials, constructed as described in Ref. 8, with semicore d electrons included in the valence configuration of both Pb and I. We use a plane-wave cutoff of 150 Ry for all ground state calculations, and $6 \times 6 \times 6$ Γ-centered $\mathbf{k}$-point mesh for the calculation of the ground state charge density.

GW calculations. All GW calculations are performed using the convergence parameters detailed in Ref. 8, reproduced here for clarity: 30 Ry exchange cutoff, 6 Ry polarizability cutoff, 1000 bands and 13.6 eV plasmon energy for the plasmon pole model.[15] A detailed convergence study for each of these parameters can be found in Ref. 8. As shown by us[8,9] and others,[16] self-consistency is important in the correct calculation of the quasiparticle band gap and effective masses of lead-halide perovskites. Here we take self-consistency into account by using a self-consistent scissor scheme described in Refs. 8, 9. Notably, in this calculation we are using a denser $\mathbf{k}$-point mesh in the calculation of the quasiparticle self-energy than that reported in Refs. 8, 9, and we obtain a band gap of 1.57 eV. The band gap is 0.14 eV smaller than that obtained in our previous calculations, while the effective masses are the same as we reported in Ref. 9.

Wannier interpolation. The Wannier interpolation follows the same steps as described in Ref. 9. We use the maximally-localized Wannier functions calculated at the DFT/LDA level to interpolate the quasiparticle eigenvalues, as described in Ref. 9. The details of this calculation are reproduced here for convenience. We consider the electronic states around the band gap, using the Pb-p and I-p orbitals as initial guesses, and extracting 72 and 24 maximally-localized Wannier functions for the valence and conduction bands, respectively. We optimize the Wannier functions for the valence and conduction band simultaneously.

Joint density of states (JDOS). For the calculation of the JDOS we use a $100 \times 100 \times 100$ uniform $\mathbf{k}$-point mesh centered at Γ to discretize the Brilloiun zone. The energy eigenvalues for each of these $\mathbf{k}$-points are obtained from Wannier interpolation. For the calculation of the JDOS we sum over all available transition energies calculated for the $100 \times 100 \times 100$ uniform $\mathbf{k}$-point mesh. Each transition is represented by a Gaussian function centered at the transition energy. The width of the transition is calculated as the imaginary

part of the electron-phonon self-energy using the EPW code,[17] as described in Ref. 18.

Independent particle absorption spectrum. We calculate the imaginary part of the independent particle relative dielectric function using the expression:[19,20]

$$\varepsilon_2(\omega) = \frac{\pi e^2 \hbar^2}{\varepsilon_0 m_{\mathrm{e}}^2 \Omega} \sum_{vc} \int \frac{d\mathbf{k}}{\Omega_{\mathrm{BZ}}} \frac{|\langle \psi_{c\mathbf{k}} | \hat{\mathbf{e}} \cdot \mathbf{p} | \psi_{v\mathbf{k}} \rangle|^2}{(\epsilon_{c\mathbf{k}} - \epsilon_{v\mathbf{k}})^2} \delta(\epsilon_{c\mathbf{k}} - \epsilon_{v\mathbf{k}} - \hbar\omega), \tag{2}$$

where e is the electronic charge, ε_0 is the dielectric permitivity of vacuum, Ω is the unit cell volume, m_{e} is the electron mass, $\hat{\mathbf{e}}$ is the polarization vector of the electromagnetic field, $\mathbf{p}$ is the electron momentum operator, $\psi_{c\mathbf{k}}$ and $\psi_{v\mathbf{k}}$ are the Kohn-Sham wave functions for the valence and conduction band states respectively, $\epsilon_{c\mathbf{k}}$ and $\epsilon_{v\mathbf{k}}$ are the corresponding Kohn-Sham eigenvalues, and $\hbar\omega$ is the photon energy. The integral is performed over the Brillouin zone and Ω_{BZ} is the Brillouin zone volume. The summation is performed over all conduction and valence band states. In our calculation we include only the first conduction band, which is sufficient to capture all optical transitions in the spectrum up to 0.6 eV above the onset.

To calculate the optical absorption using the GW band structure we neglect the off-diagonal terms of the self-energy, as described in Ref. 19, and calculate the dipole matrix elements (shown in Figure 2(c) of the main manuscript) as:[19]

$$|\langle \psi_{c\mathbf{k}} | \hat{\mathbf{e}} \cdot \mathbf{p} | \psi_{v\mathbf{k}} \rangle|^2_{\mathrm{eff}} = |\langle \psi_{c\mathbf{k}} | \hat{\mathbf{e}} \cdot \mathbf{p} | \psi_{v\mathbf{k}} \rangle|^2 \frac{(E_{c\mathbf{k}} - E_{v\mathbf{k}})^2}{(\epsilon_{c\mathbf{k}} - \epsilon_{v\mathbf{k}})^2}, \tag{3}$$

where $E_{c\mathbf{k}}$ and $E_{v\mathbf{k}}$ are the quasiparticle eigenvalues for the conduction and valence band respectively. For comparison, we calculate the GW optical absorption spectrum using either the scaled dipole matrix elements (Supplementary Equation 3) or the dipole matrix elements calculated directly from DFT, using the following two expressions:

$$\varepsilon_2^{\mathrm{GW\ scaled}}(\omega) = \frac{\pi e^2 \hbar^2}{\varepsilon_0 m_{\mathrm{e}}^2 \Omega} \sum_{vc} \int \frac{d\mathbf{k}}{\Omega_{\mathrm{BZ}}} \frac{|\langle \psi_{c\mathbf{k}} | \hat{\mathbf{e}} \cdot \mathbf{p} | \psi_{v\mathbf{k}} \rangle|^2}{(\epsilon_{c\mathbf{k}} - \epsilon_{v\mathbf{k}})^2} \delta(E_{c\mathbf{k}} - E_{v\mathbf{k}} - \hbar\omega), \tag{4}$$

$$\varepsilon_2^{\mathrm{GW\ not\ scaled}}(\omega) = \frac{\pi e^2 \hbar^2}{\varepsilon_0 m_{\mathrm{e}}^2 \Omega} \sum_{vc} \int \frac{d\mathbf{k}}{\Omega_{\mathrm{BZ}}} \frac{|\langle \psi_{c\mathbf{k}} | \hat{\mathbf{e}} \cdot \mathbf{p} | \psi_{v\mathbf{k}} \rangle|^2}{(E_{c\mathbf{k}} - E_{v\mathbf{k}})^2} \delta(E_{c\mathbf{k}} - E_{v\mathbf{k}} - \hbar\omega), \tag{5}$$

We calculate the dipole matrix elements $|\langle \psi_{c\mathbf{k}} | \hat{\mathbf{e}} \cdot \mathbf{p} | \psi_{v\mathbf{k}} \rangle|^2$ separately using the Yambo

code, and use the Wannier interpolated eigenenergies from DFT and GW, respectively, to obtain the imaginary part of the dielectric function. The optical absorption coefficient is calculated as $\alpha(\omega) = \omega\epsilon_2(\omega)/(nc)$, where we considered the refractive index to be a constant, $n = 2.4$[21] and c is the speed of light in vacuum. In all our calculations the optical matrix elements are averaged over the three polarization directions.

In order to reduce the computational cost, we calculate the optical matrix elements for a $40\times40\times40$ **k**-point mesh, using norm-conserving LDA pseudopotentials for Pb and I without the semicore d-states, and a plane wave cutoff of 50 Ry. We have checked the difference between optical matrix elements calculated with and without semicore states for 7 **k**-points along the Γ-X direction, and obtained that in the latter case the optical matrix elements are underestimated by up to 16%. In addition, all Gaussian smearings are rigidly blue-shifted by 15 meV in order to account for inhomogeneous broadening, as discussed in Ref. 18.

Effective masses. We calculate the DFT and GW effective masses as the inverse of the second derivative of the conduction and valence band edges with respect to the wave vector **k**, as described in Ref. 9. We calculate the second derivative numerically using the finite difference method as in Ref. 9, by calculating the eigenvalues at a distance of $0.01\frac{2\pi}{a}$ from Γ in reciprocal space, where a is the smallest of the three lattice parameters. In Supplementary Table 1 we show the effective masses calculated using DFT/LDA and using the GW band structure and the self-consistent scissor correction, respectively.

Supplementary Table 1: Comparison between the hole, electron and reduced effective masses calculated using DFT/LDA and the using the GW band structure and the self-consistent scissor correction. m_e is the rest electron mass.

	DFT	GW
m^*_h	$0.14\,m_\mathrm{e}$	$0.23\,m_\mathrm{e}$
m^*_e	$0.12\,m_\mathrm{e}$	$0.22\,m_\mathrm{e}$
μ	$0.06\,m_\mathrm{e}$	$0.11\,m_\mathrm{e}$

Supplementary Note 5: Modelling Of The Absorption Onset (Elliott Theory)

Supplementary Table 2: Description of variables used in the derivation of radiative bimolecular recombination.

Variable	Description
E	Energy
T	Temperature
$\otimes$	Convolution
α	Total absorption coefficient (excitons and continuum states)
α_{X}	Absorption coefficient of bound excitonic states (below-gap absorption)
α_{C}	Absorption coefficient of continuum states
α_{Free}	Absorption coefficient of free electrons and holes in the absence of Coulombic attraction
ξ	Coulombic enhancement factor
f_{Broad}	Broadening function
$\delta(E)$	Dirac delta function
E_{G}	Band gap energy
E_{X}	Exciton binding energy
$\mathrm{JDoS}(E)$	Joint density of states for transitions at photon energy E
c_0	Joint density of states constant
b_0	Proportionality constant
μ	Reduced effective mass of electron-hole system
$\mathcal{N}(u, \sigma^2)$	Normal distribution with mean u and variance σ^2
$\ln(\mathcal{N}(u, \sigma^2))$	Log-normal distribution
$\arg_E \max()$	Location of maximum
$\mathbf{w}$	Vector of local free parameters $(E_{\mathrm{G}}, E_{\mathrm{X}}, \sigma_T)$ and global parameters $(u_{\mathrm{s}}, \sigma_{\mathrm{s}})$.
y	Measured data

Our fitting procedure follows the standard least squares minimisation method:

$$S(\mathbf{w}) = \sum_{i}^{n} \left(y_i - \alpha(E_i; \mathbf{w})\right)^2 \ . \tag{6}$$

where S is the sum of the squared differences between the experimental data (y_i) and the model for the absorption coefficient (α). Our goal is to minimise S with respect to the free parameters $\mathbf{w}$ of the model. In this work we have used Elliott's model for the intensity of optical absorption by excitons.[22] The absorption model is a linear combination of the absorption from bound excitons and electron-hole continuum states and for a direct semiconductor

is written as,

$$\alpha(E) = \alpha_{\mathrm{X}}(E) + \alpha_{\mathrm{C}}(E) \tag{7}$$

with,

$$\begin{aligned}\alpha(E) = b_0 \frac{|\langle\Psi_c| P |\Psi_v\rangle|^2}{E} \Bigg(\sum_{n=1}^{\infty} \frac{4\pi E_{\mathrm{X}}^{3/2}}{n^3} \delta\left(E - \left(E_{\mathrm{G}} - \frac{E_{\mathrm{X}}}{n^2}\right)\right) \\ + \frac{2\pi\sqrt{\frac{E_{\mathrm{X}}}{E-E_{\mathrm{G}}}}}{1-\exp\left(-2\pi\sqrt{\frac{E_{\mathrm{X}}}{E-E_{\mathrm{G}}}}\right)} c_0^{-1} \mathrm{JDoS}(E) \Bigg)\end{aligned} \tag{8}$$

The joint density of states (JDoS) is given by,

$$\mathrm{JDoS}(E) = \begin{cases} c_0\sqrt{E - E_{\mathrm{G}}}, & \text{for } E > E_{\mathrm{G}} \\ 0, & \text{otherwise} \end{cases} \tag{9}$$

where,

$$c_0 = \frac{1}{(2\pi)^2}\left(\frac{2\mu}{\hbar^2}\right)^{3/2} \times 2\ . \tag{10}$$

The first term in brackets in Supplementary Equation 8 is the contribution from bound excitonic states and has the form of a line series at energies $-E_{\mathrm{X}}/n^2$ below the band gap (E_{G}) where n is a positive integer and the magnitude is proportional to $1/n^3$. A pseudo-continuum is produced as n tends to infinity, which is continuous with the absorption of continuum states at the band gap. The absorption associated with continuum states deviates from the expected square-root form for direct-gap semiconductors. Even though electrons and holes in the continuum are unbound, the Coulombic attraction enhances the absorption coefficient of these states. The Coulombic enhancement factor, ξ (equivalent to Equation 3.11 in the original article by Elliott[22] and appearing in Supplementary Equation 8) is given by:

$$\xi = \frac{2\pi\sqrt{\frac{E_{\mathrm{X}}}{E-E_{\mathrm{G}}}}}{1-\exp\left(-2\pi\sqrt{\frac{E_{\mathrm{X}}}{E-E_{\mathrm{G}}}}\right)} \tag{11}$$

It represents the probability of an electron and a hole existing in the same space and is proportional to the overlap of the electron and hole wavefunctions.[23] However, we also need to be in a position to evaluate the absorption coefficient in a system where the Coulombic attraction is not felt between electrons and holes. Physically, this can occur when the attraction is screened owing to a high background charge-carrier density in the system (i.e. above the Mott transition, as discussed further below). Turning off the Coulombic attraction is exactly equivalent to evaluating the limit of the absorption coefficient as the exciton binding energy tends towards zero, since:

$$\lim_{E_{\mathrm{X}}\to 0} \xi = 1 \tag{12}$$

Taking this limit for Supplementary Equation 8 defines the absorption coefficient $\alpha_{\mathrm{Free}}(E)$ as the value expected when Coulomb interactions are fully screened:

$$\alpha_{\mathrm{Free}}(E) \equiv \lim_{E_{\mathrm{X}}\to 0} \alpha(E) = \lim_{E_{\mathrm{X}}\to 0} \alpha_{\mathrm{C}}(E), \tag{13}$$

Hence, we obtain from Supplementary Equation 8

$$\alpha_{\mathrm{C}}(E) = \xi(E)\alpha_{\mathrm{Free}}(E) \tag{14}$$

with

$$\alpha_{\mathrm{Free}}(E) = b_0 \frac{|\langle\Psi_c|\, P\, |\Psi_v\rangle|^2}{E} c_0^{-1} \mathrm{JDoS}(E - E_{\mathrm{G}}). \tag{15}$$

Therefore, in summary, we may write our expression for the total absorption coefficient α as the sum of bound excitonic (below-gap) absorption and the free (screened) electron-hole absorption multiplied by the Coulombic enhancement factor:

$$\boxed{\alpha(E) = \alpha_{\mathrm{X}}(E) + \xi(E)\, \alpha_{\mathrm{Free}}(E)} \tag{16}$$

Supplementary Figure 4 shows the contributions of bound excitonic states (α_{X}), Coulomb-correlated continuum states (α_{C}) and free (screened, α_{Free}) electrons and holes to the total absorption coefficient spectrum $\alpha(E)$ for a range of different temperatures, obtained from

fits of Elliott Theory to the experimentally obtained absorption coefficient spectra. With decreasing temperature, excitonic effects are enhanced, leading to a larger discrepancy between α_C and α_{Free}.

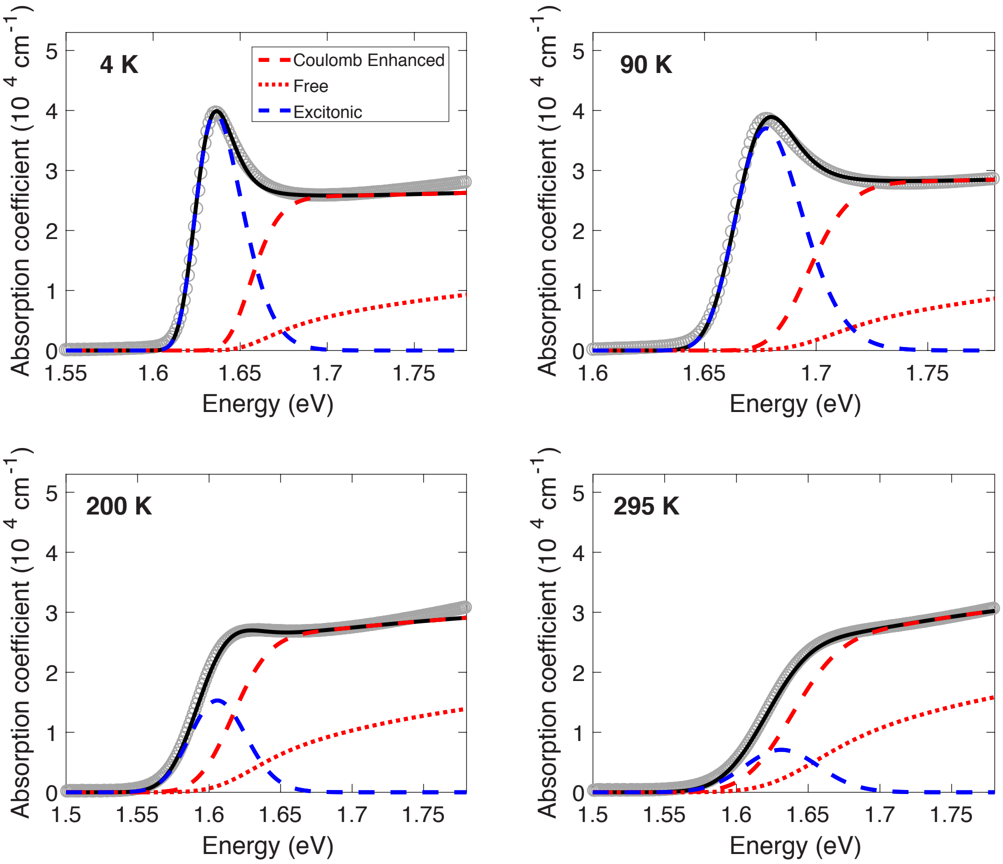

Supplementary Figure 4: Contributions to the absorption onset. Contributions of bound excitonic states (α_X), Coulomb-correlated continuum states (α_C) and free (screened, α_{Free}) electrons and holes to the total absorption coefficient spectrum $\alpha(E)$ for a range of different temperatures, obtained from fits of Elliott Theory to the experimentally obtained absorption coefficient spectra.

Electron-phonon interactions broaden the absorption, with larger effects at higher temperatures.[18] An additional energetic disorder may lead to some element of inhomogeneous broadening. We found that a log-normal distribution was useful to parameterise this additional disorder brought into the system. Previous studies have found a distribution of band gap positions varying with crystallite size and even within a single crystal.[24,25] Our model can be described as the sum of the contributions of excitonic and continuum states convolved with a broadening function. The broadening function is a normalised function centred at the maximum and is the convolution of a normal distribution, caused by electron-phonon

coupling, and a log-normal distribution, caused by disorder and local fluctuations of the stoichiometry of the material. In mathematical notation,

$$g(E) = \mathcal{N}(0,\, \sigma_T^2) \otimes \ln\big(\mathcal{N}(u_\mathrm{s},\, \sigma_\mathrm{s}^2)\big)\ , \tag{17}$$

where $\otimes$ represents a convolution, u_s and σ_s are fitted globally and σ_T are temperature dependent parameters fitted for each temperature representing the electron-phonon coupling

$$f_\mathrm{Broad}(E) = \frac{g\left(E - \arg_E \max g(E)\right)}{\int_{-\infty}^{\infty} g(E) dE}\ . \tag{18}$$

Supplementary Note 6: Calculating the Radiative Bimolecular Recombination Rate Constant (k_2) from the van-Roosbroeck and Shockley Relation

Supplementary Table 3: Description of variables used in the derivation of radiative bimolecular recombination.

Variable	Description
E	Energy
E_C	Conduction band energy level
E_V	Valence band energy level
E_F	Fermi energy level
n_0	Density of electrons in the conduction band
p_0	Density of holes in the valence band
T	Temperature
R_rad	Radiative recombination rate
k_2	Bimolecular recombination rate constant
α	Absorption coefficient
n_r	Refractive index
ρ	Equilibrium photon distribution
β	Reciprocal of the thermodynamic temperature
n_i	Intrinsic charge-carrier density
m_h^*	Effective mass of holes
m_e^*	Effective mass of electrons

Radiative recombination rate. Following the work of van Roosbroeck and Shockley,[26] which starts from the principle of detailed balance, the radiative recombination rate (R_Rad) at thermal equilibrium for an infinitesimal energy interval dE at energy E is equal to the generation rate of electron-hole pairs by thermal radiation at that energy. This rate is $P(E,T)\rho(E,T)$ per unit volume and energy interval, where $\rho(E,T)d(E)$ is the density of photons in the material in the interval dE, and $P(E,T)$ is the probability per unit time that a photon of energy E is absorbed. The total radiative recombination rate per unit volume may be written as the integral over E,

$$R_\mathrm{rad}(T) = \int_0^\infty \rho(E,T)\, P(E,T)\, dE\ . \tag{19}$$

Supplementary Equation 14 of van Roosbroeck and Shockley's article[26] gives $P(E)\rho(E)$ in

terms of frequency ν and absorption index κ,

$$P(\nu)\rho(\nu)d\nu = \frac{32\pi^2\kappa n^3}{c^3}\nu^3\frac{1}{\exp(h\nu/k_{\mathrm{B}}T)-1}d\nu \tag{20}$$

Replacing the expression for the absorption index κ with the absorption coefficient $\alpha = 4\pi n\kappa\nu/c$, and using a change of variables $h\nu = E$ gives,

$$P(E)\rho(E)dE = \frac{\alpha}{\hbar^3}\left(\frac{n_{\mathrm{r}}E}{\pi c}\right)^2\frac{1}{e^{\beta E}-1}dE\ , \tag{21}$$

where β is the reciprocal of the thermodynamic temperature. We make the approximation[27] that the refractive index in the medium is constant relative to large variations in the photon distribution ρ with temperature; $n_{\mathrm{r}} = 2.4$.[21] We consider this assumption to be reasonable since the refractive index is directly linked with absorption through the Kramers-Kronig relations.[23] We find that while the magnitude of the absorption coefficient may theoretically vary with temperature, such changes are very minor for $CH_3NH_3PbI_3$ near the band edge (see Supplementary Figure 3) suggesting that changes in refractive index are similarly negligible.

Since $e^{\beta E} \gg 1$ near the band gap, the Bose-Einstein distribution term in Supplementary Equation 21 can be written as $e^{-\beta E}$. Using this approximation we obtain the following expression for the total radiative recombination rate per unit volume:

$$R_{\mathrm{rad}}(T) = \int_0^\infty \frac{\alpha(E,T)}{\hbar^3}\left(\frac{n_{\mathrm{r}}\,E}{\pi c}\right)^2 e^{-\beta E}dE\ . \tag{22}$$

Intrinsic charge-carrier concentration. The electron density n_0 in the conduction band is given by:

$$n_0 = \int_{E_{\mathrm{C}}}^\infty \text{Electronic density of states} \times \text{Fermi-Dirac distribution } dE \tag{23}$$

$$n_0 = \int_{E_{\mathrm{C}}}^\infty \frac{2}{(2\pi)^2}\left(\frac{2m_{\mathrm{e}}^*}{\hbar^2}\right)^{3/2}\sqrt{E-E_{\mathrm{C}}} \times \frac{1}{1+e^{(E-E_{\mathrm{F}})\beta}}dE \tag{24}$$

where m_{e}^* is the effective mass for the electrons as determined from GW and shown in Supplementary Table 1, E_{C} is the conduction band energy level and E_{F} is the Fermi energy

level. For an intrinsic semiconductor, the Fermi energy level is near the middle of the band gap and hence far away from both the conduction and valence bands and so the Fermi-Dirac distribution may be approximated by the Boltzmann distributions. Hence we can write:

$$n_0 = \int_{E_C}^{\infty} \frac{2}{(2\pi)^2} \left(\frac{2m_e^*}{\hbar^2}\right)^{3/2} \sqrt{E - E_C} \times e^{-(E-E_F)\beta} dE \tag{25}$$

which is equivalent to:

$$n_0 = \frac{2}{(2\pi)^2} \left(\frac{2m_e^*}{\hbar^2}\right)^{3/2} e^{-(E_C-E_F)\beta} \int_{E_C}^{\infty} \sqrt{E - E_C} e^{-(E-E_C)\beta} dE \tag{26}$$

Using a change of variables ($x = (E - E_C)\beta$; $dx = \beta dE$):

$$n_0 = \frac{2}{(2\pi)^2} \left(\frac{2m_e^*}{\hbar^2}\right)^{3/2} e^{-(E_C-E_F)\beta} \beta^{-3/2} \int_{0}^{\infty} \sqrt{x} e^{-x} dE \tag{27}$$

The integral evaluates to $\sqrt{\pi}/2$, and so the density of electrons in the conduction band is:

$$n_0 = 2 \left(\frac{m_e^* k_B T}{2\pi\hbar^2}\right)^{3/2} e^{-(E_C-E_F)\beta} \tag{28}$$

Similarly, the density of holes p_0 can be expressed as,

$$p_0 = 2 \left(\frac{m_h^* k_B T}{2\pi\hbar^2}\right)^{3/2} e^{-(E_F-E_V)\beta} \tag{29}$$

where m_h^* is the effective mass for the holes as determined from GW and shown in Supplementary Table 1 and E_V is the valence band energy level. For an intrinsic semiconductor $n_0 = p_0 = n_i$ where n_i is the intrinsic charge-carrier density. Using $n_0 p_0 = n_i^2$ we can eliminate E_F and obtain an expression for n_i in terms of m_e^*, m_h^* and E_G (Equation 47 in Varshni's review article[27]):

$$n_i = 2 \left(\frac{k_B T}{2\pi\hbar^2}\right)^{3/2} (m_e^* m_h^*)^{3/4} e^{-\frac{1}{2}E_G\beta} \tag{30}$$

Bimolecular recombination rate. The intrinsic charge-carrier density may be used to convert the radiative recombination rate to the bimolecular recombination constant k_2,

according to

$$k_2(T) = \frac{R_{\mathrm{rad}}(T)}{n_{\mathrm{i}}(T)^2} \ . \tag{31}$$

Choice of Absorption Coefficient Used in the Calculation of k_2

The van Roosbroeck and Shockley relation requires accurate knowledge of the absorption coefficient spectrum for the calculation of the radiative recombination rate, as indicated by Supplementary Equation 22. However, as the previous section on Elliott's Theory has highlighted, careful consideration must be given to the extent to which excitonic effects are taken into account.

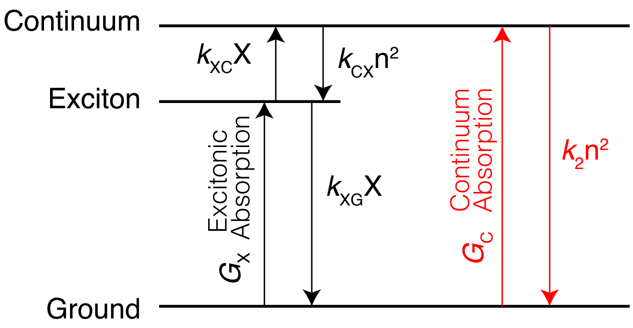

Supplementary Figure 5: Two-particle picture schematic of the energy-levels of the ground state and excited states (exciton and continuum) of a semiconductor and the respective transitions between these states.

The principle of detailed balance demands that transitions between any two states occur with equal frequency and in either direction at equilibrium and also prevents the maintenance of equilibrium by means of cyclic processes.[28] Supplementary Figure 5 shows a schematic of the energy levels for a semiconductor taking into account Coulomb correlation. For this coupled system of free carriers in the continuum states and bound electron-hole pairs in the excitonic states the rate equations are as follows:[29,30]

$$\frac{dn}{dt} = G_{\mathrm{C}} + k_{\mathrm{XC}}X - (k_{\mathrm{CX}} + k_2)n^2 \tag{32}$$

$$\frac{dX}{dt} = G_{\mathrm{X}} - (k_{\mathrm{XC}} + k_{\mathrm{XG}})X + k_{\mathrm{CX}}n^2 \tag{33}$$

where X and n are the exciton and free charge carrier densities with generation rates G_C and G_X respectively. k_XC, k_CX, and k_XG are rate constants for transitions between the ground, exciton and continuum states as detailed in Supplementary Figure 5, and k_2 is the desired rate constant of bimolecular recombination. At thermal equilibrium and with black body radiation being the only source for generation, each process must be equally balanced by the reverse process. Hence, the rate at which photons are absorbed and excite electrons and holes into the continuum states must be equally matched by the rate of emission resulting from recombination of electrons and holes in the continuum states. Similarly, the rate at which photons are absorbed and excite electrons and holes in to bound excitonic states is also equally matched by exciton recombination. Likewise, excitons dissociate to free electrons and holes in the continuum states at the same rate as formation of excitons from free electrons and holes in the continuum states. Therefore we can write:

$$G_\mathrm{C} = k_2 n_\mathrm{i}^2 = R_\mathrm{rad}^C \tag{34}$$

$$G_\mathrm{X} = k_\mathrm{XG} X_0 = R_\mathrm{rad}^X \tag{35}$$

$$k_\mathrm{XC} X_0 = k_\mathrm{CX} n_\mathrm{i}^2 \tag{36}$$

where X_0 and n_i are the equilibrium densities of excitons and free electrons and holes respectively, with n_i given by Supplementary Equation 30. The total radiative recombination will be the sum of the excitonic recombination and free electron-hole recombination to the ground state, however, only the latter is a second order process under equilibrium conditions. Hence, the absorption coefficient that should be used in the van Roosbroeck and Shockley relation in order to determine the bimolecular rate constant (k_2) is the absorption coefficient of the continuum states, with or without Coulombic enhancement, and here we discuss these two possibilities:

$\alpha_\mathbf{C}(\mathbf{E})$: The above considerations suggest that the calculation of k_2 should be based on the continuum-state absorption, but this leaves open the question of whether or not Coulomb correlations should be included. Early application of the van Roosbroeck and

Shockley relation to silicon and Groups IV, VI and III-V semiconductors[27,32–34] led to much discussion on the importance of such Coulomb effects. Schlangenotto et al.[32] concluded that calculations of the temperature-dependent bimolecular recombination rate between unbound electrons and holes must take into account Coulomb interactions in order to accurately reflect experimentally obtained values. This conclusion is intuitively sensible, since the Coulombic enhancement factor ξ increases the probability of an electron and a hole existing in the same space and therefore the bimolecular recombination rate. Therefore, in the presence of full Coulombic interactions (no screening), $\alpha_C(E)$ should be used to calculate the bimolecular recombination rate constant.

$\alpha_{\mathbf{Free}}(\mathbf{E})$**:** Use of the "free" electron-hole absorption spectrum for the calculations would assume an absence of Coulombic interactions between electrons and holes. As pointed out previously,[32] this case may be approached for charge-carrier densities above the Mott transition, for which the Coulomb interactions between carriers are effectively screened. We also note that the fully theoretical evaluation of k_2 based on DFT[35] is often limited to this option since Coulomb correlations may still be too challenging to integrate into such calculations at this point. However, such theoretical evaluation then represents the case of screened interactions at high charge-carrier densities.

In summary, calculations of k_2 to compare with experimental measurements obtained from transient spectroscopy should be based on the full (Coulomb-enhanced) continuum absorption $\alpha_C(E)$ for charge-carrier densities n below the Mott density n_M, and on the the screened absorption spectrum $\alpha_{Free}(E)$ for $n > n_M$. Supplementary Figure 6 shows the bimolecular recombination rate constants calculated from the van Roosbroeck and Shockley relation for both scenarios. Screening of Coulomb interactions leads to a clear reduction in the radiative recombination rate constant k_2 by a factor 4.4 at room temperature, but an increasing divergence is seen as the temperature is lowered. Points obtained from the absorption spectra measured at different temperatures were well represented by the following phenomenological functions (shown as black lines on the graph):

Temperature-dependent value of k_2 for Coulomb-enhanced electron-hole recom-

bination:

$$k_2(T;\alpha_\mathrm{C}) = 1.36 \times 10^{-10}\,\mathrm{cm^3 s^{-1}} \times \exp\left(\frac{702\,\mathrm{K}}{T + 49.6\,\mathrm{K}}\right) \tag{37}$$

Temperature-dependent value of k_2 for the case of screened interactions:

$$k_2(T;\alpha_\mathrm{Free}) = 0.81 \times 10^{-10}\,\mathrm{cm^3 s^{-1}} \times \exp\left(\frac{340\,\mathrm{K}}{T + 19.2\,\mathrm{K}}\right) \tag{38}$$

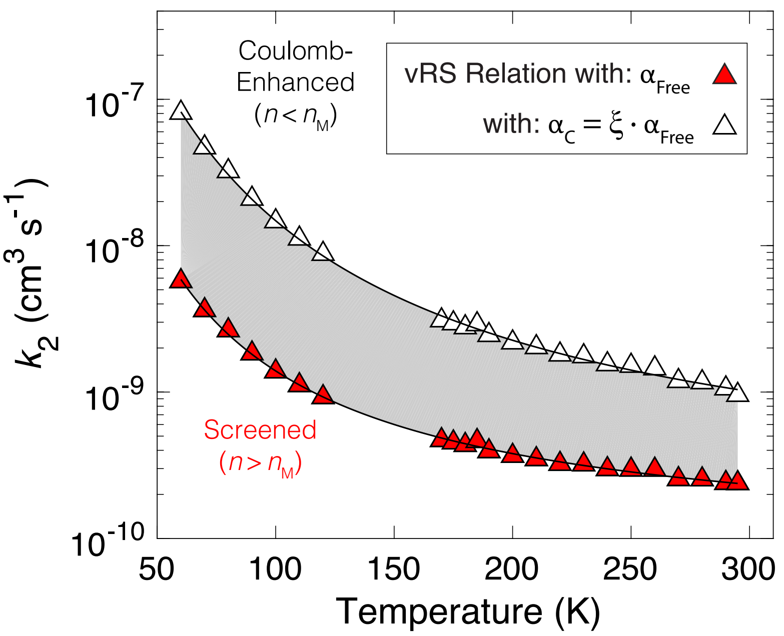

Supplementary Figure 6: Temperature dependence of bimoleulcar recombination. Bimolecular recombination rate constants k_2 calculated from the van Roosbroeck and Shockley relation, based on either the continuum-state absorption spectrum $\alpha_\mathrm{C}(E)$ as enhanced by Coulomb interactions (white triangles) and expected for charge-carrier densities below the Mott transition ($n < n_\mathrm{M}$), or the free electron-hole absorption spectrum $\alpha_\mathrm{Free}(E)$ (red triangles) for the case of screened interactions above the Mott transition ($n > n_\mathrm{M}$).

Supplementary Note 7: Mott Density

As discussed in the previous section, a correct evaluation of the bimolecular recombination rate constant depends on knowledge of the extent to which the Coulombic attraction between electrons and holes is screened. When electron and hole densities exceed the Mott density n_{M} a transition occurs from an insulating gas of excitons at lower densities to a metal-like state of an electron-hole plasma[36]. To determine whether screening effects are of importance at charge-carrier densities typically employed in transient spectroscopic measurements of k_2 we calculate the Mott densities across a wide temperature range. Previous works have reported different expressions for the Mott densities originating from model assumptions and varying degrees of model complexities. Since there is not a strong concensus in the literature on which expression best describes the transition, we report here a range of values for the Mott densities n_{M} as they emerge from different equations given in the literature as follows:[36–39]

$$n_{\mathrm{M}}^{\mathrm{Mott}} = \left(\frac{0.2}{a_{\mathrm{B}}}\right)^3 \tag{39}$$

$$n_{\mathrm{M}}^{\mathrm{Edwards}} = \left(\frac{0.26}{a_{\mathrm{B}}}\right)^3 \tag{40}$$

$$n_{\mathrm{M}}^{\mathrm{Klingshirn\text{-}1}} = 1.19^2 \frac{\epsilon_{\mathrm{r}}^{\mathrm{X}} \epsilon_0 k_{\mathrm{B}} T}{e^2 a_{\mathrm{B}}^2} \tag{41}$$

$$n_{\mathrm{M}}^{\mathrm{Klingshirn\text{-}2}} = r^{-3} \left(\frac{4}{3}\pi a_{\mathrm{B}}^3\right)^{-1} \text{ and typically } 1 < r < 5 \tag{42}$$

$$n_{\mathrm{M}}^{\mathrm{HaugSchmittRink}} = 0.028 \frac{k_{\mathrm{B}} T}{E_{\mathrm{X}} a_{\mathrm{B}}^3} \tag{43}$$

where a_{B} is the Bohr radius of the exciton, given by:

$$a_{\mathrm{B}} = a_{\mathrm{H}}\, \epsilon_{\mathrm{r}}^{\mathrm{X}} \frac{m_{\mathrm{e}}}{\mu} \tag{44}$$

with a_{H} the Bohr radius for hydrogen (5.29×10^{-11} m) and μ the reduced mass of the electron-hole system (here assumed to be 0.11 as determined from GW and shown in Supplementary Table 1).

Evaluation of Equations 39-43 and 44 also requires knowledge of the value of the dielectric function at the frequency corresponding to the exciton binding energy. We are able to determine this value of $\epsilon_{\mathrm{r}}^{\mathrm{X}}$ as a function of temperature from the exciton binding energy E_{X} (as extracted from fits based on Elliott's Theory to the absorption spectra) using:

$$\epsilon_{\mathrm{r}}^{\mathrm{X}} = \sqrt{\frac{13.6\,\mathrm{eV}}{E_{\mathrm{X}}}\frac{\mu}{m_{\mathrm{e}}}}. \tag{45}$$

Supplementary Figure 7 shows the resulting values of the exciton binding energy, Bohr radius, relevant dielectric function and Mott density as a function of temperature. Over the temperature range for which k_2 has been evaluated in this work, the Mott densities n_{M} from the above expressions are found to lie in the range of a few $10^{18}\,\mathrm{cm}^{-3}$ to $10^{16}\,\mathrm{cm}^{-3}$, with a surprisingly wide spread of possible values resulting from these different expressions. We note that this wide range mostly results from slightly different assumptions on the exact value the product $n_{\mathrm{M}}^{1/3} a_{\mathrm{B}}$ assumes in order for the Mott criterion to hold (i.e. the assessment of the distance to which excitons can approach one another before screening effects start to dominate). Since the assumed critical distance enters the various equations as a power of three, any slight changes in these assumptions can lead to very large differences in the resulting Mott density, as evident in Supplementary Figure 7d.

To assess if screening affects the values of k_2 measured in typical transient spectroscopic measurements we need to compare values stipulated for the Mott density with those at which bimolecular processes begin to feature under pulsed photoexcitation. Accurate extraction of k_2 from transient charge-carrier density decays requires the generation of charge-carrier densities for which this process begins to dominate over trap-related recombination. The charge-carrier-density dependent recombination rate is given by[31] $K(n) = k_1 + nk_2 + n^2 k_3$, where k_1, k_2 and k_3 are the rate constants for monomolecular, bimolecular and Auger processes. Hence, the onset of significant bimolecular recombination is expected to occur when

nk_2 exceeds k_1, i.e. at charge-carrier densities $\tilde{n}>(\tau k_2)^{-1}$, where $\tau=k_1^{-1}$ is the trap-related (monomolecular) recombination lifetime. For typical values τ between 10 ns and 1 μs for $MAPbI_3$[31] one obtains values for $\tilde{n}$ that range between $10^{15}\,cm^{-3}$ and $10^{17}\,cm^{-3}$. These values fall mostly below those shown in Supplementary Figure 7d although they clearly come close enough that some element of Coulomb screening may be present. In addition, we note that a typical transient spectroscopic experiment will be based on laser excitation pulses that have a spatially varying intensity profile and which generate an exponentially decaying absorption profile through the film depth, as expected from Beer's law. Therefore, a distribution of charge-carrier densities will be present, some of which may fall above, and some below the Mott density. Our considerations above therefore only serve as a rough guide, but they suggest that transient experiments sensitive to k_2 may fall into the limit below the Mott transition, for which electron-hole recombination is enhanced though Coulomb correlations, making comparison with calculations based on the van Roosbroeck and Shockley relation using the absorption coefficient of continuum states, α_C, the most viable. However, for charge-carrier denisties mostly in excess of n_M, the use of α_{Free} clearly becomes more appropriate, although changes to the absorption onset arising from band-filling effects may then also have to be included in such calculations for a fully accurate evaluation.

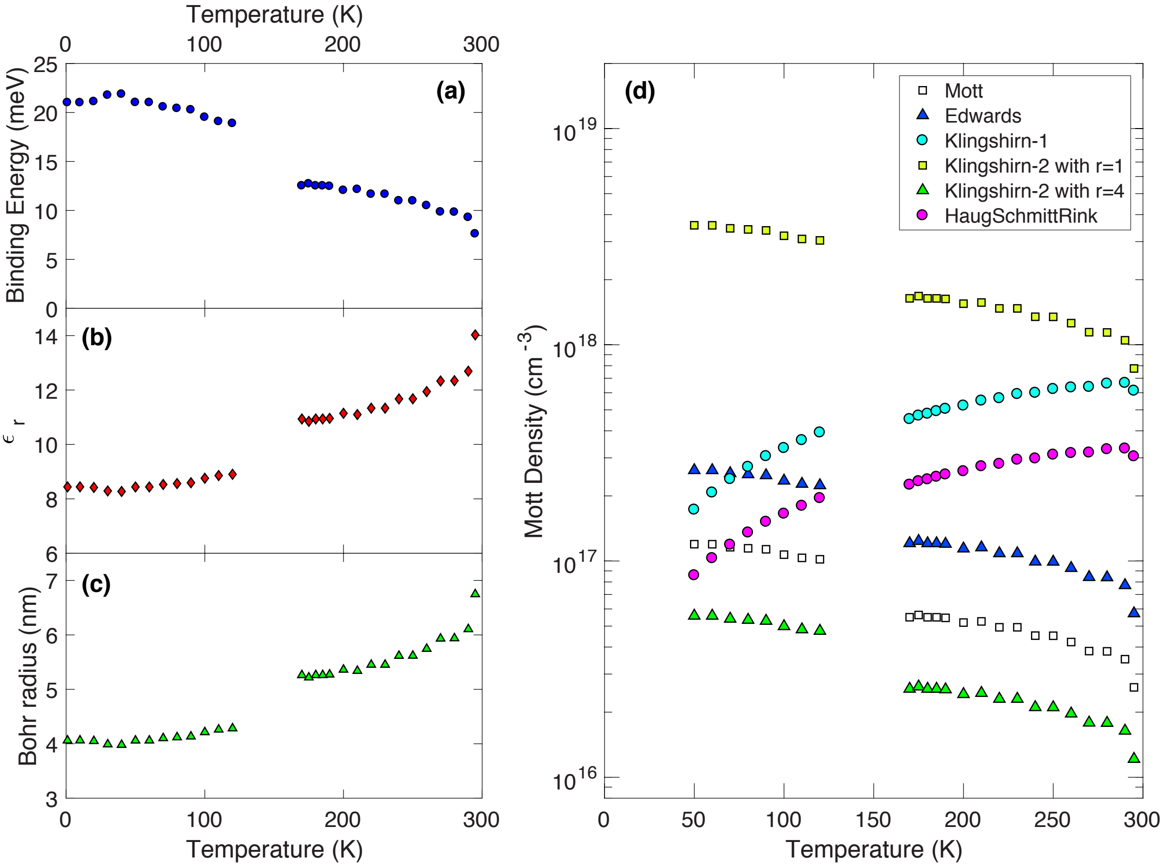

Supplementary Figure 7: Mott density derived using the extracted exciton binding energy. (a) Exciton binding energy extracted from fits of Elliott Theory to absorption spectra, which was then used for the calculation of (b) the value of the dielectric function at a frequency relevant to exciton formation (c) the exciton Bohr radius and (d) the Mott density, as described by Equations 45, 44 and 39-43 respectively.

Supplementary Note 8: Corrections to k_2 From Transient Spectroscopy to Account for Photon Re-Absorption Effects

We compare the values obtained from applying the van-Roosbroeck Shockley relation with those extracted in a previous study[40] from excitation-fluence dependent photoconductivity transients recorded after excitation with an intense short laser pulse. Such apparent bi-molecular recombination rate constants (k_2^{apparent}) however have to be corrected for self-absorption effects[41–45] to yield the intrinsic values ($k_2^{\text{intrinsic}}$). This correction accounts for the re-absorption of photoluminescence (PL) emanating from the radiative bi-molecular recombination of electrons and holes as described previously.[41] The bimolecular recombination rates obtained from applying the van-Roosbroeck Shockley relation in combination with absorption spectroscopy measurements are intrinsic values, and can therefore be directly compared with the corrected values from transient spectroscopy.

We calculate the effect of re-absorption using an explicit ray tracing model for the propagation of photons inside the perovskite. The model separates the perovskite film into a stack of slices 2 nm thick and simulates isotropic photoluminescence emission from each slice. Beer's law is used to determine the re-absorbed charge-carrier distribution in the material, and reflections at the film boundaries are governed by Fresnel equations. For a given PL wavelength, the model then calculates the fractions of PL that are re-absorbed by the perovskite, escape from the film or remain unabsorbed in the film. By averaging the results of the model across the measured PL spectrum of a sample, the overall effect of re-absorption within a sample can be determined. Supplementary Figure 8 illustrates the overlap between the measured absorption coefficient and photoluminescence spectrum at 70, 120, 170 and 295 K which causes this self-absorption effect.

To account qualitatively for re-absorption, we recorded the time-integrated photoluminescence (PL) spectra of a vapour-deposited $CH_3NH_3PbI_3$ thin film, identical to that for which absorption spectra were recorded, over temperatures from 4.4 to 295 K in increments

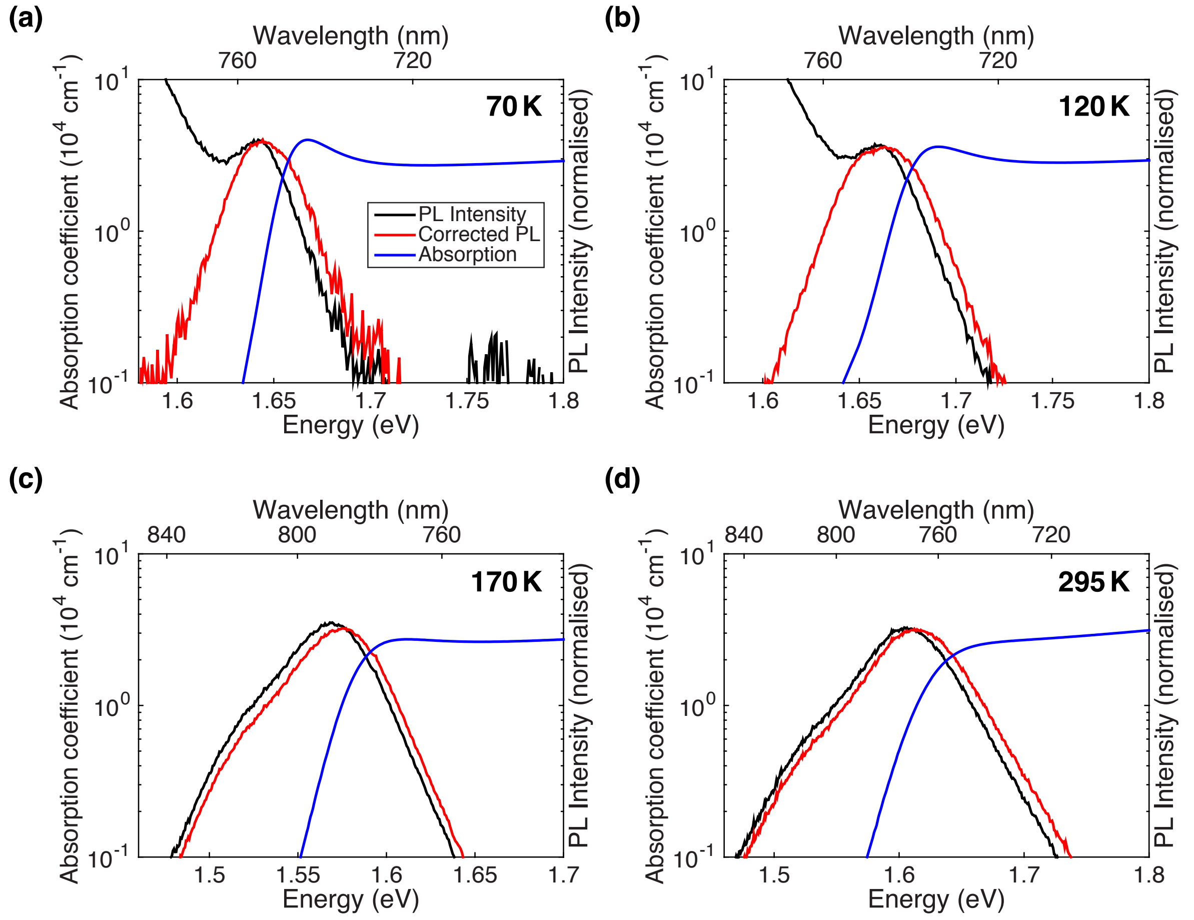

Supplementary Figure 8: Absorption coefficient and PL intensity as a function of energy of $CH_3NH_3PbI_3$ demonstrating the overlap between the two at 70, 120, 170 and 295 K for (a) to (d) respectively.

of between 5 and 10 K. The sample was mounted under vacuum ($p < 10^{-6}$ mbar) in a cold-finger liquid helium cryostat (Oxford Instruments, MicrostatHe2). An associated temperature controller (Oxford Instruments, MercuryITC) monitored the temperature at two sensors mounted on the heat exchanger of the cryostat and the end of the sample holder, respectively; the reading from the latter was taken as the sample temperature. The sample was photoexcited by a 398 nm picosecond pulsed diode laser (PicoHarp, LDH-D-C-405M) with a repetition rate of 1 MHz and a fluence of 40 nJcm^{-2}. The resultant photoluminescence (PL) was collected and coupled into a grating spectrometer (Princeton Instruments, SP-2558), which directed the spectrally dispersed PL onto an iCCD (PI-MAX4, Princeton Instruments).

Supplementary Figure 8 shows that in the tetragonal phase (> 160 K) there is one peak

resulting from band-to-band recombination. At higher temperatures, the PL peak energy blueshifts monotonically with increasing temperature, which is consistent with the known temperature dependence of the bandgap energy in hybrid lead halide perovskites.[46,47] In the orthorhombic phase ($< 160\,\mathrm{K}$) the PL spectra exhibit additional broad peaks at lower photon energies much lower than the band-gap, which has been observed many times for solution-processed $CH_3NH_3PbI_3$ thin films[18,40] and single crystals.[48] The additional emission peaks result from impurity states and small inclusions of the higher-temperature tetragonal phase that do not feature in the absorption because they are present as a minority species.[1,49] Immediately after photoexcitation, these states are therefore not populated and only become so as a result of charge-carrier diffusion[1] over the long life times of charge-carriers at the low fluences employed for these PL measurements. Hence the population of these states is only completed at times much greater than the time range probed by the transient spectroscopy measurements (2 ns) and the PL from these states will not contribute to the emitted radiation during the first 2 ns. Therefore, self-absorption over the first 2 ns after excitation is governed mostly by the PL and absorption from the majority orthorhombic phase. To account for this, we reconstruct the band-to-band recombination peak of the majority orthorhombic phase by mirroring the PL spectrum about the central peak position from the high-energy half onto the low-energy half, as displayed in Supplementary Figure 8. In addition, the PL is corrected for self-absorption effects which results in a small blue-shift in comparison with the uncorrected PL.

Using our model for PL re-absorption, we link the effective bimolecular recombination rate constants determined from transient spectroscopy[40] with the intrinsic values by determining a correction factor, $\rho = k_2^{\text{intrinsic}}/k_2^{\text{apparent}}$. This correction factor was extracted by comparing a simple charge carrier recombination model[40] used to obtain the original k_2^{apparent} and our more nuanced charge-carrier recombination model, which accounts for PL re-absorption and charge-carrier diffusion, and can determine $k_2^{\text{intrinsic}}$. By using both models to fit a range of simulated charge-carrier decays, a correction factor ρ was established as a consistent relation between the two models, shown in Supplementary Figure refPL2 and values ρ were interpolated to a required temperature that matched those at which transient spectroscopy

measurements were taken. The correction factor is specific to a given sample and is dependent on the overlap between the PL and absorption (Supplementary Figure 8), as well as the sample geometry and excitation conditions. Corrected values $k_2^{\text{intrinsic}} = \rho \times k_2^{\text{apparent}}$ are shown in Figure 3 of the main manuscript, with k_2^{apparent} taken from Ref. 40.

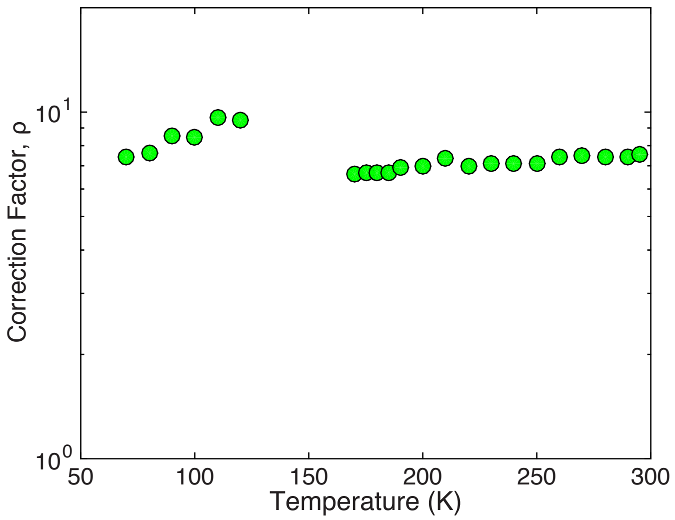

Supplementary Figure 9: Calculated correction factor $\rho = k_2^{\text{intrinsic}}/k_2^{\text{apparent}}$ as a function of temperature. This correction factor allows for transfer of bimolecular recombination rate constants derived from a simple rate equation model to values that also take into account re-absorption of photons and charge-carrier diffusion.

Supplementary References

[1] Wehrenfennig, C., Liu, M., Snaith, H. J., Johnston, M. B. & Herz, L. M. Charge carrier recombination channels in the low-temperature phase of organic-inorganic lead halide perovskite thin films. *APL Mater.* **2**, 81513 (2014).

[2] Baikie, T. *et al.* Synthesis and crystal chemistry of the hybrid perovskite ($CH_3NH_3PbI_3$) for solid-state sensitized solar applications. *J. Chem. Mater. A* **1**, 5628 (2013).

[3] Hohenberg, P. & Kohn, W. Inhomogeneous electron gas. *Phys. Rev.* **136**, B864 (1964).

[4] Perdew, J. P. & Zunger, A. Self-interaction correction to density-functional approximations for many-electrons systems. *Phys. Rev. B* **23**, 5048 (1981).

[5] Gianozzi, P. & Others. QUANTUM ESPRESSO: a modular and open-source software project for quantum simulations of materials. *J. Phys.: Condens. Matter.* **21** (2009).

[6] Hedin, L. New method for calculating the one-particle Green's function with application to the electron-gas problem. *Phys. Rev.* **139**, A796 (1965).

[7] Marini, A., Hogan, C., Grüning, M. & Varsano, D. Yambo: an ab initio tool for excited state calculations. *Comp. Phys. Commun.* **180**, 1392 (2009).

[8] Filip, M. R. & Giustino, F. GW quasiparticle band gap of the hybrid organic-inorganic perovskite $CH_3NH_3PbI_3$: effect of spin-orbit interaction, semicore electrons, and self-consistency. *Phys. Rev. B* **90**, 245145 (2014).

[9] Filip, M. R., Verdi, C. & Giustino, F. GW band structures and carrier effective masses of $CH_3NH_3PbI_3$ and hypothetical perovskites of the type $APbI_3$: A = NH_4, PH_4, AsH_4, and SbH_4. *J. Phys. Chem. C* **119**, 25209–25219 (2015).

[10] Yates, J. R., Wang, X., Vanderbilt, D. & Souza, I. Spectral and Fermi surface properties from Wannier interpolation. *Phys. Rev. B* **75**, 195121 (2007).

[11] Mostofi, A. A. *et al.* `wannier90`: A tool for obtaining maximally-localised Wannier functions. *Comp. Phys. Commun.* **178**, 685i–699 (2008).

[12] Marzari, N. & Vanderbilt, D. Maximally localized generalized Wannier functions for composite energy bands. *Phys. Rev. B* **56**, 12847 (1997).

[13] Souza, I., Marzari, N. & Vanderbilt, D. Maximally localized Wannier functions for entangled energy bands. *Phys. Rev. B* **65**, 35109 (2001).

[14] Troullier, N. & Martins, J. L. Efficient pseudopotentials for plane-wave calculations. *Phys. Rev. B* **43**, 1993 (1991).

[15] Godby, R. W. & Needs, R. J. Metal-insulator transition in Kohn-Sham theory and quasiparticle theory. *Phys. Rev. Lett.* **62**, 1169 (1989).

[16] Brivio, F., Butler, K. T., Walsh, A. & van Schilfgaarde, M. Relativistic quasiparticle self-consistent electronic structure of hybrid halide perovskite photovoltaic absorbers. *Phys. Rev. B* **89**, 155204 (2014).

[17] Ponce, S., Margine, E. R., Verdi, C. & Giustino, F. EPW: Electronphonon coupling, transport and superconducting properties using maximally localized Wannier functions. *Comp. Phys. Commun.* **209**, 116–133 (2016).

[18] Wright, A. D. *et al.* Electron-phonon coupling in hybrid lead halide perovskites. *Nat. Commun.* **7**, 11755 (2016).

[19] Del Sole, R. & Girlanda, R. Optical properties of semiconductors within the independent-quasiparticle approximation. *Phys. Rev. B* **48**, 11789–11795 (1993).

[20] Giustino, F. *Materials modelling using density functional theory* (Oxford University Press, 2014).

[21] Peréz-Osorio, M. A. *et al.* Vibrational properties of the organicinorganic halide perovskite $CH_3NH_3PbI_3$ from theory and experiment: factor group analysis, first-principles calculations, and low-temperature infrared spectra. *J. Phys. Chem. C* **119**, 25703–25718 (2015).

[22] Elliott, R. J. Intensity of optical absorption by excitons. *Phys. Rev.* **108**, 1384 (1957).

[23] Yu, P. Y. & Cardona, M. *Fundamentals of semiconductors: physics and materials properties* (Springer Science and Business Media, 2010).

[24] Draguta, S. *et al.* Spatially non-uniform trap state densities in solution-processed hybrid perovskite thin films. *J. Phys. Chem. Lett.* **7**, 715–721 (2016).

[25] Grancini, G. *et al.* Role of microstructure in the electron-hole interaction of hybrid lead-halide perovskites. *Nat. Photon.* **9**, 695–701 (2015).

[26] van Roosbroeck, W. & Shockley, W. Photon-radiative recombination of electrons and holes in germanium. *Phys. Rev.* **94**, 1558–1560 (1954).

[27] Varshni, Y. P. Band-to-band radiative recombination in groups IV, VI, and III-V semiconductors (I). *Phys. Status Solidi B* **19**, 459–514 (1967).

[28] Klein, M. J. Principle of detailed balance. *Phys. Rev.* **97**, 1446–1447 (1955).

[29] Oki, K., Ma, B. & Ishitani, Y. Population decay time and distribution of exciton states analyzed by rate equations based on theoretical phononic and electron-collisional rate coefficients. *Phys. Rev. B* **96**, 205204 (2017).

[30] Szczytko, J. *et al.* Determination of the exciton formation in quantum wells from time-resolved interband luminescence. *Phys. Rev. Lett.* **93**, 137401 (2004).

[31] Johnston, M. B. & Herz, L. M. Hybrid perovskites for photovoltaics: charge-carrier recombination, diffusion, and radiative efficiencies. *Acc. Chem. Res.* **49**, 146–154 (2016).

[32] Schlangenotto, H., Maeder, H. & Gerlach, W. Temperature dependence of the radiative recombination coefficient in silicon. *Physica Status Solidi (a)* **21**, 357–367 (1974).

[33] Ruff, M., Fick, M., Lindner, R., Rössler, U. & Helbig, R. The spectral distribution of the intrinsic radiative recombination in silicon. *Journal of applied physics* **74**, 267–274 (1993).

[34] Trupke, T. *et al.* Temperature dependence of the radiative recombination coefficient of intrinsic crystalline silicon. *Journal of Applied Physics* **94**, 4930–4937 (2003).

[35] Filippetti, A., Delugas, P. & Mattoni, A. Radiative recombination and photoconversion of methylammonium lead iodide perovskite by first principles: properties of an inorganic semiconductor within a hybrid body. *J. Phys. Chem. C* **118**, 24843–24853 (2014).

[36] Klingshirn, C. F. *Semiconductor optics* (Springer Science & Business Media, 2012).

[37] Mott, N. F. Metal-insulator transition. *Reviews of Modern Physics* **40**, 677 (1968).

[38] Edwards, P. P. & Sienko, M. J. Universality aspects of the metal-nonmetal transition in condensed media. *Physical Review B* **17**, 2575 (1978).

[39] Haug, H. & Schmitt-Rink, S. Electron theory of the optical properties of laser-excited semiconductors. *Progress in Quantum Electronics* **9**, 3–100 (1984).

[40] Milot, R. L., Eperon, G. E., Snaith, H. J., Johnston, M. B. & Herz, L. M. Temperature-dependent charge-carrier dynamics in $CH_3NH_3PbI_3$ perovskite thin films. *Adv. Funct. Mater.* **25**, 6218–6227 (2015).

[41] Crothers, T. W. *et al.* Photon reabsorption masks intrinsic bimolecular charge-carrier recombination in $CH_3NH_3PbI_3$ perovskite. *Nano Lett.* **17**, 10.1021/acs.nanolett.7b02834 (2017).

[42] Richter, J. *et al.* Enhancing photoluminescence yields in lead halide perovskites by photon recycling and light out-coupling. *Nat. Commun.* **7**, 13941 (2016).

[43] Marti, A., Balenzategui, J. L. & Reyna, R. F. Photon recycling and Shockley's diode equation. *J. Appl. Phys.* **82**, 4067–4075 (1997).

[44] Kirchartz, T., Staub, F. & Rau, U. Impact of photon recycling on the open-circuit voltage of metal halide perovskite solar cells. *ACS Energy Lett.* **1**, 731–739 (2016).

[45] Saliba, M. *et al.* Plasmonic-induced photon recycling in metal halide perovskite solar cells. *Adv. Funct. Mater.* **25**, 5038–5046 (2015).

[46] Frost, J. M. *et al.* Atomistic origins of high-performance in hybrid halide perovskite solar cells. *Nano Lett.* **14**, 2584–2590 (2014).

[47] Wright, A. D. *et al.* Band-tail recombination in hybrid lead iodide perovskite. *Adv. Funct. Mater.* (2017).

[48] Fang, H.-H. *et al.* Photophysics of organic-inorganic hybrid lead iodide perovskite single crystals. *Adv. Funct. Mater.* **25**, 2378–2385 (2015).

[49] Panzer, F. *et al.* Reversible laser induced amplified spontaneous emission from coexisting tetragonal and orthorhombic phases in hybrid lead halide perovskites. *Adv. Opt. Mater.* (2016).
